# Exosomal small RNA profiling in first-trimester maternal blood explores early molecular pathways of preterm preeclampsia

**DOI:** 10.3389/fimmu.2024.1321191

**Published:** 2024-02-22

**Authors:** Luca Gál, Ábel Fóthi, Gergő Orosz, Sándor Nagy, Nándor Gábor Than, Tamás I. Orbán

**Affiliations:** ^1^ Gene Regulation Research Group, Institute of Molecular Life Sciences, HUN-REN Research Centre for Natural Sciences, Budapest, Hungary; ^2^ Doctoral School of Biology, Institute of Biology, ELTE Eötvös Loránd University, Budapest, Hungary; ^3^ Department of Obstetrics and Gynaecology, Faculty of Medicine, University of Debrecen, Debrecen, Hungary; ^4^ Department of Obstetrics and Gynecology, Petz Aladár University Teaching Hospital, Győr, Hungary; ^5^ Faculty of Health and Sport Sciences, Széchenyi István University, Győr, Hungary; ^6^ Systems Biology of Reproduction Research Group, Institute of Molecular Life Sciences, HUN-REN Research Centre for Natural Sciences, Budapest, Hungary; ^7^ Department of Obstetrics and Gynecology, Semmelweis University, Budapest, Hungary; ^8^ Maternity Private Clinic of Obstetrics and Gynecology, Budapest, Hungary; ^9^ Genesis Theranostix Group, Budapest, Hungary

**Keywords:** biomarker, piRNA, miRNA, exosome, pregnancy, decidualization, early diagnosis, liquid biopsy

## Abstract

**Introduction:**

Preeclampsia (PE) is a severe obstetrical syndrome characterized by new-onset hypertension and proteinuria and it is often associated with fetal intrauterine growth restriction (IUGR). PE leads to long-term health complications, so early diagnosis would be crucial for timely prevention. There are multiple etiologies and subtypes of PE, and this heterogeneity has hindered accurate identification in the presymptomatic phase. Recent investigations have pointed to the potential role of small regulatory RNAs in PE, and these species, which travel in extracellular vesicles (EVs) in the circulation, have raised the possibility of non-invasive diagnostics. The aim of this study was to investigate the behavior of exosomal regulatory small RNAs in the most severe subtype of PE with IUGR.

**Methods:**

We isolated exosomal EVs from first-trimester peripheral blood plasma samples of women who later developed preterm PE with IUGR (n=6) and gestational age-matched healthy controls (n=14). The small RNA content of EVs and their differential expression were determined by next-generation sequencing and further validated by quantitative real-time PCR. We also applied the rigorous exceRpt bioinformatics pipeline for small RNA identification, followed by target verification and Gene Ontology analysis.

**Results:**

Overall, >2700 small RNAs were identified in all samples and, of interest, the majority belonged to the RNA interference (RNAi) pathways. Among the RNAi species, 16 differentially expressed microRNAs were up-regulated in PE, whereas up-regulated and down-regulated members were equally found among the six identified Piwi-associated RNAs. Gene ontology analysis of the predicted small RNA targets showed enrichment of genes in pathways related to immune processes involved in decidualization, placentation and embryonic development, indicating that dysregulation of the induced small RNAs is connected to the impairment of immune pathways in preeclampsia development. Finally, the subsequent validation experiments revealed that the hsa_piR_016658 piRNA is a promising biomarker candidate for preterm PE associated with IUGR.

**Discussion:**

Our rigorously designed study in a homogeneous group of patients unraveled small RNAs in circulating maternal exosomes that act on physiological pathways dysregulated in preterm PE with IUGR. Therefore, our small RNA hits are not only suitable biomarker candidates, but the revealed biological pathways may further inform us about the complex pathology of this severe PE subtype.

## 1 Introduction

Preeclampsia (PE) is a severe obstetrical syndrome characterized by new-onset hypertension and proteinuria after the 20th week of pregnancy. It results in severe maternal complications, such as end-organ dysfunction, and is frequently associated with fetal intrauterine growth restriction (IUGR) (1–5). Moreover, beyond the pregnancy, it can pose a life-threatening risk of cardiometabolic disease and long-term health issues for both the mother and her offspring (6, 7), and it also increases the risk of recurrence of preeclampsia in subsequent pregnancies, especially in women who have underlying immunological disorders (e.g. autoimmune diseases) (8). During pregnancy, placental development requires dynamic tissue rearrangement and remodeling of the uterine spiral arteries within the decidua and the inner third of the myometrium by a well-controlled extravillous trophoblast invasion process (9). The etiology of PE remains incompletely elucidated, but the generally accepted view is that these tightly-regulated placental developmental processes, which include trophoblast invasion, are disrupted, leading to a malfunctioning placenta, the consequent placental release of anti-angiogenic and pro-inflammatory substances, downstream maternal systemic immune cell activation, platelet activation, and disturbed fetal circulation and growth (4, 5, 10–20). PE occurring before the 34th week of pregnancy (early-onset PE, EOPE) is characterized by the insufficient development and subsequent dysfunction of the placenta, leading to strong systemic inflammation in the maternal vasculature, endothelial dysfunction, end-organ disease, and often fetal growth retardation. In contrast, the late-onset PE (LOPE) subtype, which manifests after 34 weeks of gestation, is less affected by placental disease and is strongly correlated with maternal chronic health conditions, such as pre-existing cardiovascular disease, obesity, or type 2 diabetes. It is often considered a metabolic imbalance when the placental capacity is inadequate to meet the demands of the growing fetus (2, 14, 21–28).

PE affects 2-8% of all pregnancies worldwide (7, 29), leading to the deaths of more than 75,000 women and 500,000 fetuses annually (4), but as the symptoms can usually be detected only in the second half of gestation, early detection of the disease using appropriate biomarkers remains a challenge. Recently, several studies have proposed the role of non-coding RNAs (ncRNAs) in the etiology of PE (30–33), and an emerging view is that these molecules could be transported in the circulation in membrane-encapsulated extracellular vesicles (EVs) and thus have diagnostic significance (34–40). Among the ncRNA groups, small RNAs of the RNA interference (RNAi) pathways are attractive targets for investigation because their functional roles have been more extensively elucidated as opposed to other classes, such as long ncRNAs or Y RNAs (31, 41). The most prominent representatives of the RNAi-related species are microRNAs (miRNAs) and Piwi-associated small RNAs (piRNAs). Both classes represent short single-stranded RNAs that are incorporated into Argonaute (AGO) family protein-containing RNA-induced silencing complexes (RISCs), which find their target RNA molecules through sequence complementarity (42, 43). miRNAs are shorter, typically between 20-24 nucleotides in length; they associate with the AGO-clade proteins (AGO1-4 in humans), and their effector complexes mostly target mRNAs, initiating their decay and/or inhibiting their translation (44, 45). Since a single miRNA molecule can regulate multiple target mRNAs, and *vice versa*, one mRNA can be regulated by several miRNA species, the functional role of miRNA-containing RISCs can be considered as a complex, post-transcriptional fine-tuning of gene expression (46, 47). In contrast, piRNAs are longer, with an average length of 27-35 nucleotides, are 2’-OH methylated at their 3’ end, and associate with the PIWI clade of AGO proteins (PIWIL1-4 in humans) (48). They were originally considered to be the “guardians” of germline cells mainly by targeting transposable elements (49–51), but recent studies have also uncovered several functions of piRNAs in somatic cells (48, 52).

Recently, numerous investigations have been published describing the putative roles of various small RNAs in PE, but the overlap among these predicted targets is typically small (33, 53–59). Apart from technical differences, a major concern with these investigations is that the included patient groups in these studies were not generally uniform due to the inherent heterogeneity of the disease. As described earlier, PE can be divided into at least two major subtypes based on the clinical onset of the symptoms (EOPE versus LOPE), but various further subclasses can be defined based on the patients’ molecular profiles (2, 22, 24, 60–62). Therefore, to find reliable etiological factors and potential biomarkers, this disease heterogeneity must be considered in the study design. Here, we aimed to investigate the early molecular background of PE at the small RNA level in a well-defined, clinically homogeneous population of patients with the most severe phenotype, which develops before 37 weeks of gestation and strongly affects fetal growth.

## 2 Materials and methods

### 2.1 Study population, clinical definition

Patient recruitment and sample collection were carried out during the second phase of the Hungarian Perinatal Study (HUN-PER) at the Department of Obstetrics and Gynecology of the Petz Aladár County Teaching Hospital in Győr, and at the Department of Obstetrics and Gynecology of the University of Debrecen in Debrecen.

First-trimester plasma samples from six Caucasian women who later developed PE associated with IUGR and 14 healthy controls matched for gestational age (GA) within one week of sample collection were selected for inclusion. GA was determined based on fetal crown-rump length (CRL) measured by ultrasound scan between the 10th and 13th weeks of pregnancy. PE was defined as new-onset hypertension developing after 20 weeks of gestation (systolic and/or diastolic blood pressure of >140mmHg and/or >90mmHg, respectively, measured on at least 2 occasions, 4 hours to 1 week apart) coupled with proteinuria (>300mg in a 24-hour urine collection or 2 random urine specimens with ≥1+ protein by dipstick collected 4 hours to 1 week apart or one random urine specimen with ≥2+ protein by dipstick) (63). Early-onset PE was defined as PE that developed at <34 weeks of gestation (64). IUGR was defined as fetal weight either below the 3rd percentile or below the 10th percentile combined with Doppler anomalies (65). Women in the control group had an uncomplicated pregnancy which resulted in the delivery of an appropriate-for-gestational-age neonate at term (>37 weeks of gestation).

### 2.2 Sample collection and handling

Venous blood samples were collected between the 10th and 13th weeks of pregnancy in 4 mL EDTA tubes and kept at 4°C for a maximum of 3 hours prior to processing. Plasma was separated by centrifuging blood (2,000×g, 10min, 4°C followed by 10,000×g, 10min, 4°C), and then stored in 300μl aliquots at −80°C. The samples and the associated clinical and demographic information, including the delivery and medication records, were stored anonymously at the Perinatal Biobank of the Research Centre for Natural Sciences in Budapest, Hungary (https://www.perinatalbiobank.com/).

### 2.3 Exosome and RNA extraction

For the procedure, we followed the previously published protocol (66). Briefly, 300μl aliquots of stored plasma samples were centrifuged at 3000×g for 5 minutes, then pre-filtered through a 0.22 μm syringe filter to remove cell debris and larger-sized EVs. Exosomes were isolated from ~200 μl of pre-filtered plasma samples and subsequently, total RNA was extracted from the exosomes using the exoRNeasy Midi Kit (QIAGEN) following the manufacturer’s instructions.

### 2.4 Small RNA sequencing

Small RNA sequencing was performed by Lexogen GmbH without bioinformatics or subsequent analysis. RNA integrity was assessed on a Fragment Analyzer System using the DNF-471 RNA Kit (Agilent). Multiplexed sequencing-ready indexed small RNA libraries were prepared using the Small RNA-Seq Library Prep Kit for Illumina (052UG128V0110) following the procedure as described in the User’s Guide. For library preparation, an average of 1 ng of the total RNA sample was used as an input without small RNA enrichment. The quality control of the library preparation was checked by HS DNA assay for the Fragment Analyzer system (Agilent). The concentration of the resulting libraries was quantified using a Qubit dsDNA HS assay (Thermo Fisher). Next-Generation Sequencing (NGS) was performed on the Illumina NextSeq 2000 platform.

Bioinformatics analysis was performed using the exceRpt pipeline (version 4.3.2) (67). Read Per Million (RPM) normalized readcounts were used for the analysis of differential expression in exosomal small RNA, employing the glmQL method within the edgeR package (version 3.38.4) (68). The raw and processed sequencing data are available in the GSE241815 dataset at Gene Expression Omnibus (GEO).

### 2.5 Target prediction and gene ontology

The target mRNAs of the miRNAs were predicted by the miRabel web tool with a miRabel score of less than 0.05. The target genes of piRNAs were identified by miRanda 3.3a against the human transcriptome (hg38) with a maximum free energy of -20 kcal/mol and a miRanda score threshold of 160. Only protein-coding targets were subjected to subsequent analyses. Gene Ontology (GO) analysis was performed using the web tool ShinyGo 0.77. When the target mRNAs of miRNAs and piRNAs were handled separately, the target mRNAs of a minimum of 4 miRNAs and a minimum of 2 piRNAs were selected for GO. When the target mRNAs of up- and down-regulated small RNAs were examined, the genes targeted by a minimum of 5 up-regulated or 1 down-regulated small RNA were selected for GO.

### 2.6 Reverse-transcription and quantitative real-time PCR

cDNA synthesis was performed using the miRCURY LNA RT Kit (QIAgen), with 0.56 μl of RNA templates added to each reaction according to the equation “Template RNA [µl] = Elution volume [µl]/Original sample volume [µl] * 8 [µl]” (https://www.qiagen.com/us/resources/resourcedetail?id=34039664-5bf4-42b1-9858-f4c28dace788&lang=en). qPCR was carried out using the miRCURY Probe PCR Kit, run on a StepOnePlus™ platform (Thermo Fisher Scientific) according to the manufacturer’s instructions. miRCURY LNA miRNA Probe Assays were applied in the case of hsa-miR-122-5p, while custom-made miRCURY LNA miRNA Custom Probe Assays were used in the case of hsa_piR_016658 (**Table 1**). During qPCR measurements, we applied the ΔΔCt method. We used hsa-miR-21-5p as an endogenous control and we normalized the small RNA expression levels to the mean expression levels of the control samples instead of using a dedicated reference sample.

**Table 1:** Sequences of mature small RNAs investigated by qPCR.

small RNA name	small RNA sequence
**hsa-miR-21-5p**	5’-UAGCUUAUCAGACUGAUGUUGA-3’
**hsa-miR-122-5p**	5’-UGGAGUGUGACAAUGGUGUUUG-3’
**hsa_piR_016658**	5’-CCCCCCACUGCUAAAUUUGACUGGCUA-3’

### 2.7 Statistical analyses

We used R 4.2.2 for statistical analysis. The *Shapiro-Wilk test* was carried out to determine the normality of the continuous variables in the patient descriptive table. For normally distributed continuous variables, we assessed the homogeneity of variance using the *F-test*, and subsequently, we used the *two sample t-test* to examine statistical significance. For non-normally distributed continuous variables, we used the non-parametric *Wilcoxon Rank-Sum test* for comparison. For categorical variables, *Fisher’s exact test* was used for comparison between groups. In the sequencing data analysis, the differential exosomal small RNA expression was identified by the *quasi-likelihood F-test*, where the GA at sampling (in weeks) calculated by CRL was added as an additional experimental factor in the design matrix. -ΔΔCt values of the small RNAs were represented on box plots as medians with interquartile ranges. *p-values* were calculated by the logistic regression model, in which we adjusted for GA at sampling (in weeks). Results were considered statistically significant at a *p-value* of <0.05.

## 3 Results

### 3.1 Characteristics of the study population

To investigate the changes in exosomal small RNA content associated with PE in early pregnancy, we selected first-trimester maternal plasma samples from 14 control individuals and 6 patients with PE associated with IUGR from our biobank. The demographic and clinical characteristics of the study groups are shown in **Table 2**. Due to the strict gestational age matching, GA values at sampling were not different between the groups. However, several parameters showed significant differences, including blood pressure, proteinuria, GA at delivery, birth weight, and birth weight percentile. Interestingly, we could also find prior allergies in two-thirds of the PE patients, which is in accord with a previous epidemiological study that revealed maternal allergy as an isolated risk factor for early-onset preeclampsia (69), suggesting an immunological origin of this severe subtype of PE.

**Table 2:** Clinical and demographic characteristics of the study groups.

Parameters	Control (n = 14*^1^*)	PE + IUGR (n = 6*^1^*)	p-value*^2^*
**Systolic BP (mmHg)** ¤	112 ± 10	152 ± 14	<0.001
**Diastolic BP (mmHg)** ¤	71 ± 5	90 ± 4	<0.001
**Proteinuria** #	0/14 (0%)	5/6 (83.3%)	<0.001
**GA at delivery (week)** *	39.4 ± 0.8	32.7 ± 4.1	<0.001
**Mode of conception**			
Spontaneous	14/14 (100.0%)	6/6 (100.0%)	
**Type of delivery** #			0.050
C-section	4/14 (28.6%)	5/6 (83.3%)	
Spontaneous	10/14 (71.4%)	1/6 (16.7%)	
**CRL (mm)** *	37 ± 13	37 ± 15	0.8
**GA at time of sampling (week)** *	10.14 ± 1.03	10.00 ± 1.26	0.7
**Newborn weight (g)** ¤	3474 ± 444	1433 ± 626	<0.001
**EFW percentile** *	51 ± 28	1 ± 0	<0.001
**Newborn sex** #			0.6
Boy	7/14 (50.0%)	2/6 (33.3%)	
Girl	7/14 (50.0%)	4/6 (66.7%)	
**Ethnicity**			
Caucasian	14/14 (100.0%)	6/6 (100.0%)	
**Maternal age (years)** ¤	31 ± 4	35 ± 5	0.055
**BMI (kg/m²)** ¤	23.2 ± 3.2	25.4 ± 5.0	0.3
**Gravidity** *	2.43 ± 1.22	1.83 ± 1.60	0.2
**Parity** *	1.29 ± 1.07	0.67 ± 0.82	0.2
**Nulliparity** #	3/14 (21.4%)	3/6 (50.0%)	0.3
**Maternal history of PE** #	0/14 (0%)	2/6 (33.3%)	0.079
**Family history of PE** #	0/14 (0%)	1/6 (16.7%)	0.3
**Allergy **#	2/14 (14.3%)	4/6 (66.7%)	0.037
**Gestational diabetes** #	0/14 (0%)	1/6 (16.7%)	0.3
**Medication at the time of sampling** #	0/14 (0%)	1/6 (16.7%)	0.3
**Smoking status** #	0/14 (0%)	1/6 (16.7%)	0.3

^1^Mean ± SD; n/N (%).

^2^Two sample t-test (¤); Wilcoxon rank sum test (*); Fisher’s exact test (#). BMI – body mass index, BP – blood pressure, CRL – crown-rump length, EWF – estimated fetal weight, GA – gestational age, IUGR – intrauterine growth restriction, PE – preeclampsia.

Demographic and clinical parameters of the study groups were presented as either mean and standard deviation (SD) or frequencies. Neither chronic diseases (such as chronic diabetes or chronic hypertension) nor autoimmune disorders (including Systemic Lupus Erythematosus or Antiphospholipid Syndrome) were diagnosed, therefore they are not included in the table. Appropriate statistical analyses were performed according to the distribution types of the data. Statistically significant differences between the control and the PE+IUGR groups were accepted at the level of p<0.05.

### 3.2 Changes in exosomal small RNA profiles of PE patients in the discovery samples

**Figure 1:**
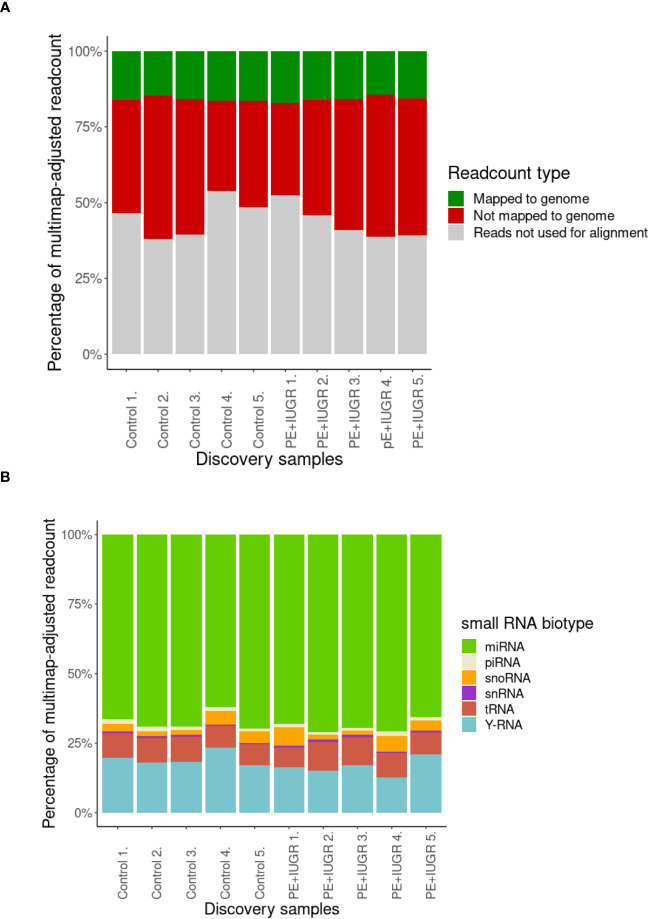
Analysis of first-trimester maternal plasma-derived exosomal small RNA sequencing data from the discovery samples in the PE (n=5) and control (n=5) groups. **(A)** Percentage of mappable and unmappable reads, and those that could not be used for multimap-adjusted alignment. **(B)** Percentage of small RNA biotypes in maternal plasma-derived exosomal samples.

For small RNA analysis, exosomes were isolated from blood plasma samples of 5 randomly selected control and 5 PE patients, representing “discovery samples”. Following total RNA isolation from the exosomes, the samples were sequenced for small RNAs and the results were analyzed using the bioinformatics pipeline described in the Materials and Methods section. Although limited by the small amounts of starting material, and thereby, the total number of reads, similar proportions of the reads could be mapped to the human genome in all samples (**Figure 1A**). Since the majority of small RNAs in all samples belong to miRNAs (**Figure 1B**), we decided to focus on the RNA interference pathways in further analyses, and therefore, also included the detected piRNAs in our study.

**Figure 2:**
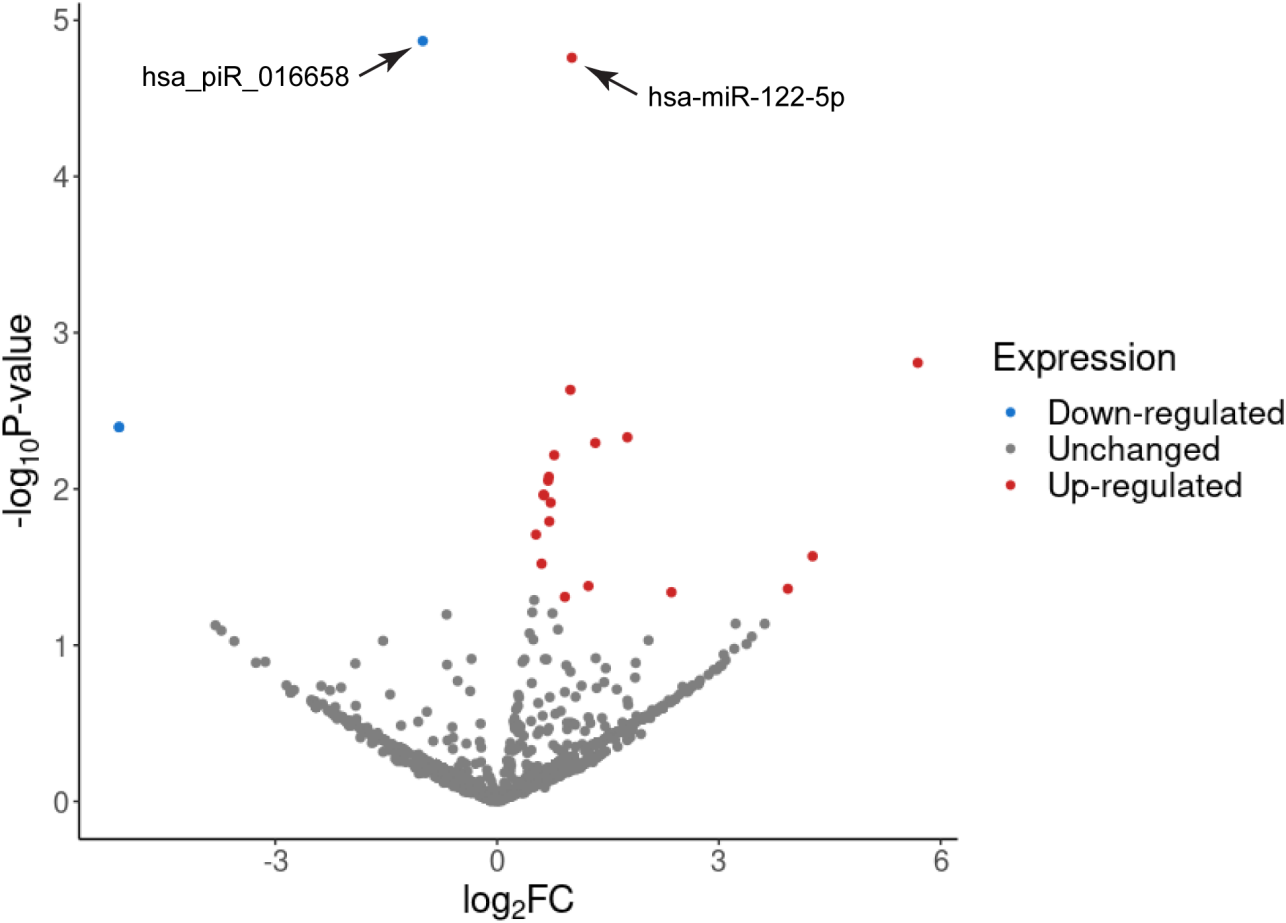
Volcano plot of the differentially expressed exosomal miRNAs and piRNAs in PE associated with IUGR. Differentially expressed small RNAs were counted using the edgeR quasi-likelihood F-test with gestational week at the time of sampling as an independent variable. The x-axis represents the log2 transformed ratio of expression between the PE and control groups and the y-axis shows the -log10 of the p-values. Small RNAs validated by qPCR are depicted by arrows.

GA is associated with changes in the expression levels of different kinds of molecules in the maternal circulation, such as miRNAs and proteins (70, 71). Therefore, GA at the time of sampling was included as an additional variable (covariant) in our analyses to calculate the differential gene expression levels. The adjusted expression values were then used to determine the differential miRNA and piRNA expression profiles as shown in **Figure 2**. We found a total of 22 small RNA species that showed significant differential expression in samples from PE pregnancies as compared to the control group (**Table 3**). The majority of these small RNAs were up-regulated in PE-derived exosomes, including 16 miRNAs and 3 piRNAs (**Table 3**), whereas only 3 piRNAs were found to be down-regulated (with negative log_2_FC values in **Table 3**). Two of these down-regulated piRNAs (hsa_piR_001331 and hsa_piR_000577) had exactly the same gene expression values which raises the possibility that they may belong to the same piRNA cluster.

**Table 3:** Differentially expressed exosomal small RNAs in preterm PE with IUGR.

small RNA ID	log_2_FC	p-value	FDR
**hsa_piR_016658**	-1.01	0.00001	0.01118
**hsa-miR-122-5p**	1.01	0.00002	0.01118
**hsa-miR-4535-3p**	5.69	0.00156	0.66835
**hsa_piR_021675**	0.99	0.00232	0.74667
**hsa_piR_001331**	-5.11	0.00402	0.81659
**hsa_piR_000577**	-5.11	0.00402	0.81659
**hsa-miR-20a-5p**	1.76	0.00467	0.81659
**hsa-miR-302a-5p**	1.33	0.00508	0.81659
**hsa-miR-144-3p**	0.77	0.00608	0.85661
**hsa-miR-143-3p**	0.70	0.00837	0.85661
**hsa-miR-183-5p**	0.69	0.00885	0.85661
**hsa-miR-185-5p**	0.63	0.01088	0.85661
**hsa-miR-186-5p**	0.63	0.01101	0.85661
**hsa-miR-1-3p**	0.72	0.01223	0.85661
**hsa-miR-501-3p**	0.71	0.01613	0.85661
**hsa-miR-30a-5p**	0.53	0.01959	0.85661
**hsa-miR-125b-2-3p**	4.27	0.02695	0.85661
**hsa-miR-96-5p**	0.60	0.03008	0.85661
**hsa-miR-144-5p**	1.23	0.04182	0.85661
**hsa_piR_018007**	3.93	0.04360	0.85661
**hsa_piR_020249**	2.36	0.04582	0.85661
**hsa-miR-26b-5p**	0.92	0.04911	0.85661

List of the differentially expressed exosomal miRNAs and piRNAs in PE associated with IUGR by ascending p-values, also showing the log2-transformed fold change (FC) and false discovery rate (FDR) values. All 16 miRNAs and 3 piRNAs were up-regulated, while 3 piRNAs were down-regulated.

### 3.3 Target analyses of differentially expressed exosome-derived small RNAs

**Figure 3:**
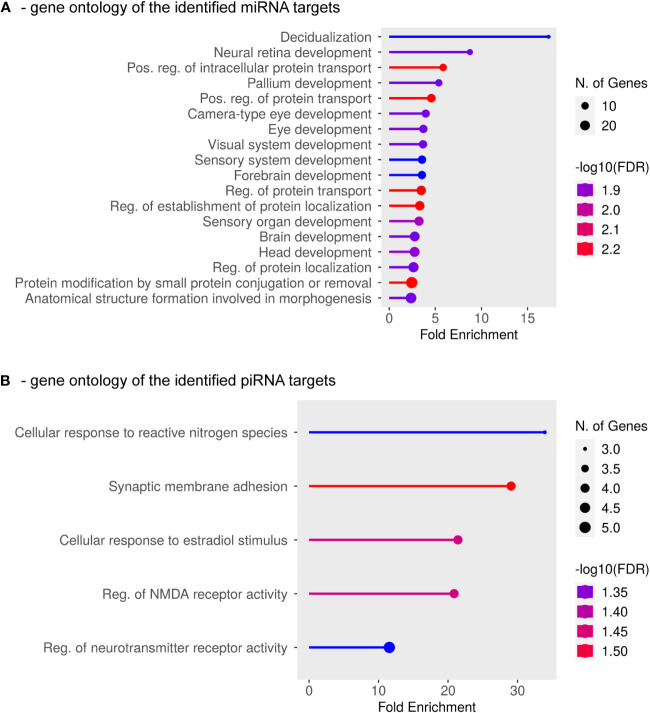
Gene Ontology of mRNAs targeted by differentially expressed exosomal miRNAs and piRNAs. GO Biological Process Analysis of 219 mRNAs targeted by a minimum of four miRNAs **(A)** and 118 mRNAs targeted by a minimum of two piRNAs **(B)**. The complete list of the analyzed mRNAs is shown in **Supplementary Table 1**.

The function of both miRNAs and piRNAs is to regulate mRNA targets mostly at the post-transcriptional level, therefore, we carried out a bioinformatics analysis to search for potential mRNAs targeted by the differentially expressed small RNAs. To apply stringent prediction criteria for miRNA targets, we considered those transcripts of protein-coding genes that contain a minimum of four miRNA binding sites (of the same or different miRNA species). By performing Gene Ontology (GO) analysis, we found that among the 219 predicted targets (the complete list of identified target mRNAs is in **Supplementary Table 1**), the genes with the highest enrichment are, for example, those involved in ‘decidualization’ (**Figure 3A**), an important biological process that ensures the adequate implantation of the embryo and maternal-fetal immune interactions during pregnancy (72, 73). Indeed, ‘embryo implantation’ and ‘placental development’ were also among the impacted biological processes similar to the ‘regulation of blood vessel endothelial cell migration’, all of which are disrupted in preterm severe PE as discussed above. In addition, the majority of the other enrichment categories represent various developmental pathways (such as nervous system development) that are required for normal embryogenesis. These results thus strongly indicate that the identified exosomal miRNAs are crucial for the regulation of normal pregnancy, implantation, and embryonic development, and are likely to be targeted to both maternal and fetal tissues, with their dysregulation closely linked to maternal and fetal pathogenesis in preterm severe PE.

For piRNAs, target prediction is still precarious, as the details of the effector function of piRNA-loaded RISCs in higher organisms, especially in human tissues, are currently under investigation (51, 74). Based on several models (75, 76), we decided to use the miRanda platform to identify mRNA targets with at least two potential piRNA binding sites. Subsequent GO analysis of the identified 118 target mRNAs showed enrichments in nitrogen stress response, estradiol-regulated pathways, and certain neuronal functions, all of which are related to perinatal developmental processes (**Figure 3B**), further supporting the relevance of the identified piRNAs in the regulation of critical developmental processes in pregnancy.

**Figure 4:**
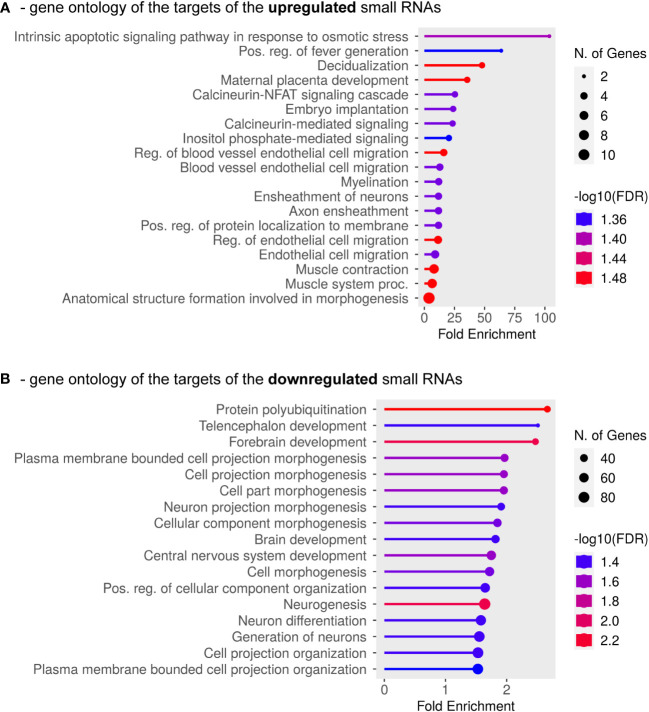
GO Biological Process Analysis was carried out for mRNAs targeted by the identified down-regulated or up-regulated exosomal small RNAs. **(A)** Results are shown for mRNAs targeted by a minimum of five up-regulated small RNAs (miRNAs and/or piRNAs). **(B)** GO categories are shown for mRNAs targeted by at least one down-regulated small RNA (only piRNAs were found to be down-regulated in this study). The complete list of the analyzed mRNAs is shown in **Supplementary Table 2**.

Having focused on whether any changes in small RNA expression profiles are connected to PE, in a subsequent investigation we asked whether the directionality of the expression changes is important. We considered all miRNAs and piRNAs together that were either up-regulated or down-regulated and analyzed the GO categories predicted for their target mRNAs (the complete list is in **Supplementary Table 2**). The protein-coding transcripts targeted by the up-regulated small RNAs were especially enriched in the osmotic stress response pathway, but the subsequent categories were also strongly related to pregnancy or placental disorders (decidualization or placental development, blood vessel endothelial remodeling), as well as to calcineurin-NFAT signaling, inositol-phosphate-mediated signaling, embryo implantation and developmental processes (**Figure 4A**). When analyzing the GO classes of the mRNAs targeted by the down-regulated small RNA species (in this case, piRNAs only), significant enrichments were identified in protein polyubiquitination and various cellular morphogenesis pathways, especially those related to neurodevelopmental processes (**Figure 4B**). Taken together, all analyses indicated that the identified small RNA species play important roles in normal placental formation and embryonic development, and their dysregulation is strongly connected to disease progression in severe preterm PE associated with IUGR.

### 3.4 Validation of the identified small RNA expression profiles in an expanded sample set

**Figure 5:**
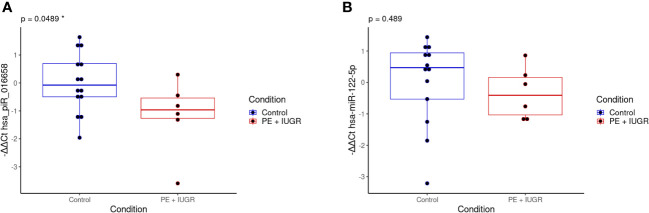
qPCR validation of the selected differentially expressed exosomal small RNAs in the validation samples. The box plots show the exosomal expression levels of hsa_piR_016658 **(A)** and hsa-miR-122-5p **(B)** small RNAs for the PE (n=6, red) and control (n=14, blue) samples. -ΔΔCt values are shown, and black dots represent normalized small RNA expression levels for each individual patient. The corresponding p-values are shown at the top of each graph and were calculated by a logistic regression model in which the completed gestational weeks at the time of sampling were used as an independent variable. *: statistical significance at p<0.05.

To validate the small RNA sequencing results, we selected small RNAs with FDR<0.05 values (**Table 3**) and measured their expression levels by qPCR methodology. The limited number of clinical samples restricted our measurements to a study population of six PE and 14 control samples which also included the discovery samples. The correlation between the GA at the time of sampling and the small RNA exosomal expression levels examined by qPCR was also remarkable (**Supplementary Figure 1**), so we included the gestational weeks at the time of sampling as an independent variable in our model. As shown in **Figure 5**, the expression level of the hsa_piR_016658 showed a significant difference between PE patients and controls. In contrast, the expression of the selected hsa-miR-122-5p miRNA did not show a significant difference between the two groups.

## 4 Discussion

### 4.1 Principal findings of the study

1) In total, more than 2700 small RNAs were identified in all samples, and of interest, the majority belong to the RNAi pathways. 2) Among the RNAi species, 16 differentially expressed microRNAs were up-regulated in PE, whereas up-regulated and down-regulated members were equally found among the six identified Piwi-associated RNAs. 3) Gene ontology analysis of the predicted small RNA targets showed enrichment of genes in pathways related to immune processes involved in decidualization, placentation, and embryonic development, indicating that the dysregulation of the elicited small RNAs is connected to the impairment of immune pathways in the development of preeclampsia. 4) The subsequent validation experiments revealed that the hsa_piR_016658 piRNA is a promising candidate biomarker for preterm PE associated with IUGR.

### 4.2 A homogeneous group of preterm PE patients with IUGR and severe immune disease is our focus

Previous investigations frequently neglected the inherent variability among PE subtypes (77) and the molecular markers elicited in several studies often showed little overlap. Therefore, here we aimed to examine a clearly defined preterm severe subtype of PE that is also associated with fetal growth restriction. This homogenous group of women had pro-inflammatory conditions before pregnancy, including prior maternal allergies (in two-thirds of PE patients), which is in agreement with a previous epidemiological study identifying this risk factor for early-onset PE (75), and underlying a potential immunological background of this severe PE subtype. Although the rigorous selection criteria clearly limited the number of includable samples, this clinically homogeneous group held promise for the identification of pathophysiologically relevant differences. At this early stage of pregnancy, the placenta is not fully developed, and our results may represent the early steps of PE pathogenesis and indicate very early diagnostic markers. In addition to the strict gestational age matching between disease and control samples, we also applied the completed gestational weeks at the time of sampling as a covariant in our bioinformatic models to minimize the bias potentially introduced by gestational age-related changes in exosomal and small RNA quantities in maternal blood.

### 4.3 Preterm PE-related circulating exosomal small RNAs are associated with placental disease pathways

We focused strictly on the small RNA content of circulating exosomes, aiming to discover novel types of potential biomarkers for this life-threatening PE subtype. It was intriguing to see that the majority of small RNA reads were mapped to miRNAs, and the most abundant species showing no significant expression difference between the groups was hsa-miR-486-5p, which has been widely shown to be present in exosomes (78–80). The miRNAs showing significantly different expression in exosomes were all up-regulated (**Table 3**) and the majority of them have already been described in connection with various pregnancy-related disorders. These included hsa-miR-1-3p (81), hsa-miR-183-5p (82, 83), hsa-miR-185-5p (84), hsa-miR-186-5p (85), and hsa-miR-20a-5p (81), and the latter three are also normally expressed at higher levels in the first trimester of healthy pregnancies (86), although in certain studies where exosomes were not separated they are referred to as plasma-specific miRNAs (87). Interestingly, hsa-miR-26b-5p showed a tendency for upregulation in PE, but was not found to be differentially expressed in another study (88), which may be due to the heterogeneous patient population included in that study. The hsa-miR-30a-5p was also found to be up-regulated in PE (89) but it was also described as the most abundant cell-free miRNA in the urine (90), raising the possibility of using this biofluid for PE investigations in the future. In contrast to our findings, there were some miRNA species that exhibited reduced expression profiles in placental disorders, such as hsa-miR-143-3p (81) and hsa-miR-96-5p (91). On the other hand, hsa-miR-122-5p showed a remarkable variation: it was found up-regulated in one study (88) but down-regulated in a later one (92), and this variability currently remains unexplained. Of interest, the status of hsa-miR-125b-2-3p is difficult to assess in recent publications because it cannot be determined with certainty which arms or isomiRs were measured in the studies (81, 93, 94). A similar question on miRNA arm usage also applies to the hsa-miR-302a or the hsa-miR-144 loci: in both cases, the downregulation of the 3p arm was detected in PE samples (88, 95), as compared to our data on the upregulation of the 5p arm (**Table 3**). Although tissue-specific miRNA arm selection cannot be ruled out (47), it is intriguing that for both loci, the miRNA database lists the 5p arms as the dominant ones (https://mirbase.org/). However, the differences compared to our study may arise from the comparison of placental tissue results with exosomal results, as well as from the clinical heterogeneity within the PE group (81). Finally, there are a number of previously found miRNAs (96) that we could not associate with PE. In some cases, such as for the members of the placenta-specific C19MC miRNA cluster, this is due to the early gestation samples when the placenta is less developed; in other cases, certain miRNAs may be predominantly present in the serum but not in the exosomes, such as the hsa-miR-146b-5p (97). On the other hand, it is of interest that we could identify two miRNA species, hsa-miR-501-3p and hsa-miR-4535-3p, which have not yet been associated with PE, suggesting further studies in relation to the whole PE syndrome, or even to different PE subclasses.

In our study, we also detected overexpressed and underexpressed exosomal piRNAs in PE samples. When searching for previously published data, none of these piRNA hits were associated with placental disorders or PE; in fact, we could find published data concerning only one of the piRNAs, namely hsa_piR_016658. Apart from generally being detected in body fluids (90, 98) and even in EVs (99), this piRNA was also found to be overexpressed in the prostate (100) and endometrial cancer (78), or after hypoxia in adipose-derived stromal cells and stem cells (101). Of interest, we detected decreased exosomal expression of this piRNA which may reflect the increased oxygen concentrations in the placental blood spaces or the ischemic environment at the maternal-fetal interface in early pregnancy in patients who subsequently developed PE (102, 103).

Another remarkable finding in our study was that the two other down-regulated piRNAs, namely hsa_piR_001331 and hsa_piR_000577, had exactly the same expression values, suggesting a possible co-regulation, perhaps by being part of the same piRNA cluster. We have found that hsa_piR_001331 and hsa_piR_000577 share a 25-nucleotide-long overlapping region at five different genomic locations (3p26.33 sense, 5q12.1 sense, 5p14.2 anti-sense, 7p21.17 anti-sense and 15q21.2 anti-sense); however, hsa_piR_001331 has 38 genomic copies, whereas hsa_piR_000577 is represented by only six copies in the genome (http://pirnabank.ibab.ac.in/). These findings could support the claim that the piRNAs are in the same cluster, but this would require further analysis since members of small RNA clusters can also be regulated individually (104, 105).

### 4.4 Circulating exosomal small RNAs are associated with immune processes during decidualization, implantation and throughout pregnancy

An important aspect of validation is the analysis of the potential target genes of the small RNA hits. At first, we treated miRNAs and piRNAs separately, considering any change in expression, regardless of its direction (**Figure 3**). When analyzing the GO classifications, the identified miRNA targets were particularly enriched in genes associated with decidualization (**Supplementary Table 1**), some of which have already been implicated in placental disorders. Among them, cyclooxygenase-2 (COX-2, also known as PTGS2) is up-regulated in the first trimester in healthy pregnancies and its deficiency leads to the loss of implantation and decidualization, as well as to PE or to preterm birth (106, 107). Prostacyclin, an end product of COX-2, is formed during inflammation and is a key target of non-steroidal anti-inflammatory drugs such as aspirin, the most widely used preventive treatment to reduce the incidence of the most severe preterm PE subtype (108–112). Stanniocalcin-1 (STC-1) is a pleiotropic hormone that is important for maintaining female reproductive health and shows a sharp placental expression peak in mid-gestation (113), and its increased mRNA level has been detected in pregnancy complications such as PE or gestational diabetes mellitus (114, 115). Another important target gene is the transmembrane protein connexin-43 (GJA-1), which is vital for direct intracellular communication and also in placental development and trophoblast differentiation by supporting appropriate vasculature in the placental bed, and its dysregulation has been suggested to have a potential role in the development of PE (116, 117).

### 4.5 Differentially expressed piRNAs may regulate nitrative and oxidative stress responses in PE

The top GO category for mRNA targets of differentially expressed piRNAs was ‘cellular response to reactive nitrogen species’. Abnormal trophoblast invasion into the uterine spiral arteries leads to increased ischemia and pro-inflammatory changes in the placenta, resulting in oxidative and nitrative stress and leading to the accumulation of nitrotyrosine, a potential biomarker associated with PE and IUGR (118). mRNA hits from our analysis revealed key players in the oxidative and nitrative stress responses. These include the CASP8 and FADD Like Apoptosis Regulator (*CFLAR*), an essential activator of the extrinsic pathway of apoptosis (119), and the PPARG Coactivator 1 Alpha (*PPARGC1A*), a master regulator of mitochondrial biogenesis and antioxidant defense (120), with decreased protein levels in the placenta in PE, especially in cases associated with IUGR (121). In addition, we also identified the Methionine synthase (*MTR*) gene which had an increased expression in PE and is clearly associated with the compensation of methionine-homocysteine metabolism caused by oxidative stress in this obstetrical syndrome (122, 123).

### 4.6 Exosomal small RNAs are involved in additional vascular and immunological processes

In a subsequent analysis, we grouped the mRNA targets based on either upregulation or downregulation of the small RNA hits (treating miRNAs and piRNAs together, **Figure 4**). Here, the GO categories are enriched in decidualization, placental development, and embryo implantation, and the top target genes show a high overlap with the previous analysis above, including *COX2*, *GJA1* and *STC1*. In addition, however, we found three members of the calcineurin-NFAT signaling cascade and inositol-phosphate-mediated signaling pathways that play central roles in different aspects of appropriate immune responses such as T-cell activated adaptive immune response (124) and in B cell immunity (125). The identified targets are the ATPase plasma membrane Ca2+ transporter (*ATP2B4*, also known as *PMCA4*), the Protein Phosphatase 3 Regulatory Subunit B Alpha (*PPP3R1*, also known as *CNB1*), and the Homer Scaffold Protein 2 (*HOMER2*), all of which are involved in T-cell regulation (126–128). Moreover, in relation to pregnancy disorders, *ATP2B4* had decreased mRNA and protein levels in syncytiotrophoblasts cultured from preeclamptic placental tissue (129), whereas *PPP3R1* mRNA levels were increased in PE-associated placentas (130).

Finally, we could identify gene hits from the GO category of the ‘blood vessel endothelial cell migration’ pathway: these included the already-found *ATP2B4* and *COX2* genes, but also Neurofibromin1 (*NF1*), a gene mutated in neurofibromatosis 1 patients, who have a higher risk of developing PE and IUGR (131). The correlation of the identified GO categories and target genes with the examined PE disease subtype supports the relevance of the identified small RNA species in the circulating exosomes. However, further studies are needed to investigate whether these exosomal miRNAs and piRNAs can in fact reach relevant tissues (e.g. placenta, endothelial cells, or even embryo-derived cells) and whether the revealed target mRNAs are regulated by these delivered small RNAs in the specific cell types.

## 5 Summary and conclusion

Through comparative analysis of normal and preterm PE pregnancies, we could identify several exosomal miRNAs and piRNAs as potential early biomarkers for this severe PE subtype. Due to the strict selection criteria, we could analyze a relatively small number of cases but the stringent bioinformatic analysis and GO classification results validated our rigorous approach and showed a clear connection between these RNA expression levels and placental dysfunction in PE. Although the samples examined represent the first trimester of pregnancy when the placenta is not yet fully developed, the importance and clear advantage is that the small RNA profiles revealed may still indicate the early steps of the pathogenesis at a time when the clinical symptoms of PE cannot yet be detected using the currently accepted medical examinations. However, the identified small RNAs, especially the most promising hsa_piR_016658, would still require further validation in a larger number of patients. In addition, our approach to exosomal isolation and analysis may be problematic if the available blood sample volumes are limited; from this aspect, specific qPCR analysis directly on blood samples may be more practical, or the analysis of other body fluids (such as urine samples) could be considered. On the other hand, future studies should also focus on the target genes of the recently identified small RNAs and the applicability of other small RNA subtypes, such as Y-RNAs or tRNA species, which could also be detected in our sequencing analysis but their detailed analysis was beyond the scope of our current study.

In conclusion, our rigorously designed study yielded meaningful results from a small but homogeneous patient group. The differentially expressed small RNAs in circulating maternal exosomes act on physiological pathways involved in normal decidualization, placentation, maternal-fetal immune interactions, and fetal development, all of which are disrupted in preterm PE associated with IUGR. Therefore, our small RNA hits are not only suitable biomarker candidates for future investigations, but the revealed biological pathways may also inform us about the complex pathology during the development of this very severe subtype of PE. Furthermore, the small RNA species identified, together with their potential targets could contribute to the development of new treatment possibilities, especially for women in the early stages of pregnancy.

## Data availability statement

The datasets presented in this study can be found in online repositories. The names of the repository/repositories and accession number(s) can be found below: GSE241815 (GEO).

## Ethics statement

Clinical samples and data collection were approved by the Health Science Board of Hungary (ETT-TUKEB 4834-0/2011-1018EKU). The studies were conducted in accordance with the local legislation and institutional requirements. The participants provided their written informed consent to participate in this study. Specimens and data were stored anonymously. 

## Author contributions

LG: Conceptualization, Data curation, Formal analysis, Funding acquisition, Investigation, Methodology, Software, Validation, Visualization, Writing – original draft, Writing – review & editing. ÁF: Data curation, Investigation, Methodology, Software, Writing – review & editing. GO: Methodology, Resources, Writing – review & editing. SN: Methodology, Resources, Writing – review & editing. NGT: Conceptualization, Data curation, Funding acquisition, Investigation, Methodology, Supervision, Validation, Writing – review & editing. TIO: Conceptualization, Data curation, Formal analysis, Funding acquisition, Investigation, Methodology, Project administration, Supervision, Validation, Writing – original draft, Writing – review & editing.

## Supplementary material

Supplementary Figure 1 Correlation between gestational weeks at sampling time and exosomal small RNA expression measured by qPCR for **(A)** hsa_piR_016658, *p-value*=0.051, and **(B)** hsa-miR-122-5p, *p-value*=0.122, for PE (n=6, red) and control (n=14, blue) samples.

## References

[1] Duley L. The global impact of pre-eclampsia and eclampsia. Semin Perinatol. (2009) 33:130–7. doi: 10.1053/j.semperi.2009.02.010 19464502

[2] Tranquilli AL Dekker G Magee L Roberts J Sibai BM Steyn W. The classification, diagnosis and management of the hypertensive disorders of pregnancy: A revised statement from the ISSHP. Pregnancy Hypertens. (2014) 4:97–104. doi: 10.1016/j.preghy.2014.02.001 26104417

[3] Burton GJ Redman CW Roberts JM Moffett A. Pre-eclampsia: pathophysiology and clinical implications. BMJ. (2019) 366:l2381. doi: 10.1136/bmj.l2381 31307997

[4] Steegers EA von Dadelszen P Duvekot JJ Pijnenborg R. Pre-eclampsia. Lancet. (2010) 376:631–44. doi: 10.1016/S0140-6736(10)60279-6 20598363

[5] Chaiworapongsa T Chaemsaithong P Yeo L Romero R. Pre-eclampsia part 1: current understanding of its pathophysiology. Nat Rev Nephrol. (2014) 10:466–80. doi: 10.1038/nrneph.2014.102 PMC5893150 25003615

[6] Myatt L Roberts JM. Preeclampsia: syndrome or disease? Curr Hypertens Rep. (2015) 17:83. doi: 10.1007/s11906-015-0595-4 26362531

[7] Chaemsaithong P Sahota DS Poon LC. First trimester preeclampsia screening and prediction. Am J Obstet Gynecol. (2022) 226:S1071–S97 e2. doi: 10.1016/j.ajog.2020.07.020 32682859

[8] D'Ippolito S Barbaro G Paciullo C Tersigni C Scambia G Di Simone N. Antiphospholipid syndrome in pregnancy: new and old pathogenetic mechanisms. Int J Mol Sci. (2023) 24:3195. doi: 10.3390/ijms24043195 36834614 PMC9966557

[9] Burton GJ Woods AW Jauniaux E Kingdom JC. Rheological and physiological consequences of conversion of the maternal spiral arteries for uteroplacental blood flow during human pregnancy. Placenta. (2009) 30:473–82. doi: 10.1016/j.placenta.2009.02.009 PMC2697319 19375795

[10] Redman CWG Staff AC Roberts JM. Syncytiotrophoblast stress in preeclampsia: the convergence point for multiple pathways. Am J Obstet Gynecol. (2022) 226:S907–S27. doi: 10.1016/j.ajog.2020.09.047 33546842

[11] Lyall F Robson SC Bulmer JN. Spiral artery remodeling and trophoblast invasion in preeclampsia and fetal growth restriction: relationship to clinical outcome. Hypertension. (2013) 62:1046–54. doi: 10.1161/HYPERTENSIONAHA.113.01892 24060885

[12] Sacks GP Studena K Sargent K Redman CW. Normal pregnancy and preeclampsia both produce inflammatory changes in peripheral blood leukocytes akin to those of sepsis. Am J Obstet Gynecol. (1998) 179:80–6. doi: 10.1016/s0002-9378(98)70254-6 9704769

[13] Hahn S Giaglis S Hoesli I Hasler P. Neutrophil NETs in reproduction: from infertility to preeclampsia and the possibility of fetal loss. Front Immunol. (2012) 3:362. doi: 10.3389/fimmu.2012.00362 23205021 PMC3506920

[14] Robillard PY Dekker G Scioscia M Saito S. Progress in the understanding of the pathophysiology of immunologic maladaptation related to early-onset preeclampsia and metabolic syndrome related to late-onset preeclampsia. Am J Obstet Gynecol. (2022) 226:S867–S75. doi: 10.1016/j.ajog.2021.11.019 35177223

[15] Miller D Motomura K Galaz J Gershater M Lee ED Romero R. Cellular immune responses in the pathophysiology of preeclampsia. J Leukoc Biol. (2022) 111:237–60. doi: 10.1002/JLB.5RU1120-787RR PMC8511357 33847419

[16] Erez O Romero R Espinoza J Fu W Todem D Kusanovic JP. The change in concentrations of angiogenic and anti-angiogenic factors in maternal plasma between the first and second trimesters in risk assessment for the subsequent development of preeclampsia and small-for-gestational age. J Matern Fetal Neonatal Med. (2008) 21:279–87. doi: 10.1080/14767050802034545 PMC2846114 18446652

[17] Espinoza J Romero R Mee Kim Y Kusanovic JP Hassan S Erez O. Normal and abnormal transformation of the spiral arteries during pregnancy. J Perinat Med. (2006) 34:447–58. doi: 10.1515/JPM.2006.089 PMC7062302 17140293

[18] Tarca AL Romero R Benshalom-Tirosh N Than NG Gudicha DW Done B. The prediction of early preeclampsia: Results from a longitudinal proteomics study. PloS One. (2019) 14:e0217273. doi: 10.1371/journal.pone.0217273 31163045 PMC6548389

[19] Chaiworapongsa T Romero R Kusanovic JP Mittal P Kim SK Gotsch F. Plasma soluble endoglin concentration in pre-eclampsia is associated with an increased impedance to flow in the maternal and fetal circulations. Ultrasound Obstet Gynecol. (2010) 35:155–62. doi: 10.1002/uog.7491 PMC2944768 20101637

[20] Nagy B Sulyok E Varnagy A Barabas A Kovacs K Bodis J. The role of platelets in reproduction. Orv Hetil. (2022) 163:1254–60. doi: 10.1556/650.2022.32530 35933620

[21] Mavreli D Lykoudi A Lambrou G Papaioannou G Vrachnis N Kalantaridou S. Deep sequencing identified dysregulated circulating microRNAs in late onset preeclampsia. In Vivo. (2020) 34:2317–24. doi: 10.21873/invivo.12044 PMC7652460 32871756

[22] Brown MA Magee LA Kenny LC Karumanchi SA McCarthy FP Saito S. The hypertensive disorders of pregnancy: ISSHP classification, diagnosis & management recommendations for international practice. Pregnancy Hypertens. (2018) 13:291–310. doi: 10.1016/j.preghy.2018.05.004 29803330

[23] Bokslag A van Weissenbruch M Mol BW de Groot CJ. Preeclampsia; short and long-term consequences for mother and neonate. Early Hum Dev. (2016) 102:47–50. doi: 10.1016/j.earlhumdev.2016.09.007 27659865

[24] Raymond D Peterson E. A critical review of early-onset and late-onset preeclampsia. Obstet Gynecol Surv. (2011) 66:497–506. doi: 10.1097/OGX.0b013e3182331028 22018452

[25] Vokalova L van Breda SV Ye XL Huhn EA Than NG Hasler P. Excessive neutrophil activity in gestational diabetes mellitus: could it contribute to the development of preeclampsia? Front Endocrinol (Lausanne). (2018) 9:542. doi: 10.3389/fendo.2018.00542 30298053 PMC6161643

[26] Sharma S Banerjee S Krueger PM Blois SM. Immunobiology of gestational diabetes mellitus in post-medawar era. Front Immunol. (2021) 12:758267. doi: 10.3389/fimmu.2021.758267 35046934 PMC8761800

[27] Robillard PY Dekker G Scioscia M Bonsante F Boukerrou M Iacobelli S. Preeclampsia in 2023: Time for preventing early onset- and term preeclampsia: The paramount role of gestational weight gain. J Reprod Immunol. (2023) 158:103968. doi: 10.1016/j.jri.2023.103968 37290173

[28] Szalai G Romero R Chaiworapongsa T Xu Y Wang B Ahn H. Full-length human placental sFlt-1-e15a isoform induces distinct maternal phenotypes of preeclampsia in mice. PloS One. (2015) 10:e0119547. doi: 10.1371/journal.pone.0119547 25860260 PMC4393117

[29] Sibai B Dekker G Kupferminc M. Pre-eclampsia. Lancet. (2005) 365:785–99. doi: 10.1016/S0140-6736(05)17987-2 15733721

[30] Jelena M Sopic M Joksic I Zmrzljak UP Karadzov-Orlic N Kosir R. Placenta-specific plasma miR518b is a potential biomarker for preeclampsia. Clin Biochem. (2020) 79:28–33. doi: 10.1016/j.clinbiochem.2020.02.012 32092293

[31] Munjas J Sopic M Stefanovic A Kosir R Ninic A Joksic I. Non-coding RNAs in preeclampsia-molecular mechanisms and diagnostic potential. Int J Mol Sci. (2021) 22:10652. doi: 10.3390/ijms221910652 34638993 PMC8508896

[32] Kondracka A Jaszczuk I Koczkodaj D Kondracki B Fraszczak K Oniszczuk A. Analysis of circulating C19MC microRNA as an early marker of hypertension and preeclampsia in pregnant patients: A systematic review. J Clin Med. (2022) 11:7051. doi: 10.3390/jcm11237051 36498625 PMC9739231

[33] He J Chen M Xu J Fang J Liu Z Qi H. Identification and characterization of Piwi-interacting RNAs in human placentas of preeclampsia. Sci Rep. (2021) 11:15766. doi: 10.1038/s41598-021-95307-w 34344990 PMC8333249

[34] Awoyemi T Cerdeira AS Zhang W Jiang S Rahbar M Logenthiran P. Preeclampsia and syncytiotrophoblast membrane extracellular vesicles (STB-EVs). Clin Sci (Lond). (2022) 136:1793–807. doi: 10.1042/CS20220149 PMC9751756 36511102

[35] Geekiyanage H Rayatpisheh S Wohlschlegel JA Brown R Jr. Ambros V. Extracellular microRNAs in human circulation are associated with miRISC complexes that are accessible to anti-AGO2 antibody and can bind target mimic oligonucleotides. Proc Natl Acad Sci USA. (2020) 117:24213–23. doi: 10.1073/pnas.2008323117 PMC7533700 32929008

[36] Barranco I Salas-Huetos A Berlanga A Spinaci M Yeste M Ribas-Maynou J. Involvement of extracellular vesicle-encapsulated miRNAs in human reproductive disorders: a systematic review. Reprod Fertil Dev. (2022) 34:751–75. doi: 10.1071/RD21301 35527383

[37] Gallo DM Fitzgerald W Romero R Gomez-Lopez N Gudicha DW Than NG. Proteomic profile of extracellular vesicles in maternal plasma of women with fetal death. J Matern Fetal Neonatal Med. (2023) 36:2177529. doi: 10.1080/14767058.2023.2177529 36813269 PMC10395052

[38] Nagy B Csanadi Z Poka R. The importance of "free" nucleic acids in the non-invasive diagnostics. Orv Hetil. (2016) 157:1900–9. doi: 10.1556/650.2016.30621 27889980

[39] Nagy B. Cell-free nucleic acids in prenatal diagnosis and pregnancy-associated diseases. EJIFCC. (2019) 30:215–23. PMC6599189 31263394

[40] Biro O Fothi A Alasztics B Nagy B Orban TI Rigo J Jr. Circulating exosomal and Argonaute-bound microRNAs in preeclampsia. Gene. (2019) 692:138–44. doi: 10.1016/j.gene.2019.01.012 30659946

[41] Veziroglu EM Mias GI. Characterizing extracellular vesicles and their diverse RNA contents. Front Genet. (2020) 11:700. doi: 10.3389/fgene.2020.00700 32765582 PMC7379748

[42] Ghildiyal M Zamore PD. Small silencing RNAs: an expanding universe. Nat Rev Genet. (2009) 10:94–108. doi: 10.1038/nrg2504 19148191 PMC2724769

[43] Iwakawa HO Tomari Y. Life of RISC: Formation, action, and degradation of RNA-induced silencing complex. Mol Cell. (2022) 82:30–43. doi: 10.1016/j.molcel.2021.11.026 34942118

[44] Gebert LFR MacRae IJ. Regulation of microRNA function in animals. Nat Rev Mol Cell Biol. (2019) 20:21–37. doi: 10.1038/s41580-018-0045-7 30108335 PMC6546304

[45] Huntzinger E Izaurralde E. Gene silencing by microRNAs: contributions of translational repression and mRNA decay. Nat Rev Genet. (2011) 12:99–110. doi: 10.1038/nrg2936 21245828

[46] Arora S Rana R Chhabra A Jaiswal A Rani V. miRNA-transcription factor interactions: a combinatorial regulation of gene expression. Mol Genet Genomics. (2013) 288:77–87. doi: 10.1007/s00438-013-0734-z 23334784

[47] Orban TI. One locus, several functional RNAs-emerging roles of the mechanisms responsible for the sequence variability of microRNAs. Biol Futur. (2023) 74:17–28. doi: 10.1007/s42977-023-00154-7 36847925

[48] Wang X Ramat A Simonelig M Liu MF. Emerging roles and functional mechanisms of PIWI-interacting RNAs. Nat Rev Mol Cell Biol. (2022) 24:123–41. doi: 10.1038/s41580-022-00528-0 36104626

[49] Onishi R Yamanaka S Siomi MC. piRNA- and siRNA-mediated transcriptional repression in Drosophila, mice, and yeast: new insights and biodiversity. EMBO Rep. (2021) 22:e53062. doi: 10.15252/embr.202153062 34347367 PMC8490990

[50] Peng JC Valouev A Liu N Lin H. Piwi maintains germline stem cells and oogenesis in Drosophila through negative regulation of Polycomb group proteins. Nat Genet. (2016) 48:283–91. doi: 10.1038/ng.3486 PMC4767590 26780607

[51] Ozata DM Gainetdinov I Zoch A O'Carroll D Zamore PD. PIWI-interacting RNAs: small RNAs with big functions. Nat Rev Genet. (2019) 20:89–108. doi: 10.1038/s41576-018-0073-3 30446728

[52] Peng JC Lin H. Beyond transposons: the epigenetic and somatic functions of the Piwi-piRNA mechanism. Curr Opin Cell Biol. (2013) 25:190–4. doi: 10.1016/j.ceb.2013.01.010 PMC3651849 23465540

[53] Jaszczuk I Winkler I Koczkodaj D Skrzypczak M Filip A. The role of cluster C19MC in pre-eclampsia development. Int J Mol Sci. (2022) 23:13836. doi: 10.3390/ijms232213836 36430313 PMC9699419

[54] Xu P Ma Y Wu H Wang YL. Placenta-derived microRNAs in the pathophysiology of human pregnancy. Front Cell Dev Biol. (2021) 9:646326. doi: 10.3389/fcell.2021.646326 33777951 PMC7991791

[55] Ntsethe A Mackraj I. An Investigation of Exosome Concentration and Exosomal microRNA (miR-155 and miR-222) Expression in Pregnant Women with Gestational Hypertension and Preeclampsia. Int J Womens Health. (2022) 14:1681–9. doi: 10.2147/IJWH.S382836 PMC9741850 36514348

[56] Martinez-Fierro ML Garza-Veloz I. Analysis of circulating microRNA signatures and preeclampsia development. Cells. (2021) 10:1003. doi: 10.3390/cells10051003 33923172 PMC8145322

[57] Zou G Ji Q Geng Z Du X Jiang L Liu T. miR-31-5p from placental and peripheral blood exosomes is a potential biomarker to diagnose preeclampsia. Hereditas. (2022) 159:35. doi: 10.1186/s41065-022-00250-z 36123601 PMC9484067

[58] Salomon C Guanzon D Scholz-Romero K Longo S Correa P Illanes SE. Placental exosomes as early biomarker of preeclampsia: potential role of exosomal microRNAs across gestation. J Clin Endocrinol Metab. (2017) 102:3182–94. doi: 10.1210/jc.2017-00672 28531338

[59] Devor E Santillan D Scroggins S Warrier A Santillan M. Trimester-specific plasma exosome microRNA expression profiles in preeclampsia. J Matern Fetal Neonatal Med. (2020) 33:3116–24. doi: 10.1080/14767058.2019.1569614 PMC7328825 30700172

[60] Than NG Posta M Gyorffy D Orosz L Orosz G Rossi SW. Early pathways, biomarkers, and four distinct molecular subclasses of preeclampsia: The intersection of clinical, pathological, and high-dimensional biology studies. Placenta. (2022) 125:10–9. doi: 10.1016/j.placenta.2022.03.009 PMC9261837 35428514

[61] Tamas P Betlehem J Szekeres-Bartho J Kovacs K Wami GA Vertes V. The two faces of preeclampsia. Orv Hetil. (2022) 163:663–9. doi: 10.1556/650.2022.32427 35462351

[62] Than NG Romero R Gyorffy D Posta M Bhatti G Done B. Molecular subclasses of preeclampsia characterized by a longitudinal maternal proteomics study: distinct biomarkers, disease pathways and options for prevention. J Perinat Med. (2023) 51:51–68. doi: 10.1515/jpm-2022-0433 36253935 PMC9837387

[63] Bulletins–Obstetrics ACoP. ACOG practice bulletin. Diagnosis and management of preeclampsia and eclampsia. Obstet Gynecol. (2002) 99:159–67. doi: 10.1016/s0029-7844(01)01747-1. Number 33, January 2002. 16175681

[64] von Dadelszen P Magee LA Roberts JM. Subclassification of preeclampsia. Hypertens Pregnancy. (2003) 22:143–8. doi: 10.1081/PRG-120021060 12908998

[65] Melamed N Baschat A Yinon Y Athanasiadis A Mecacci F Figueras F. FIGO (international Federation of Gynecology and obstetrics) initiative on fetal growth: best practice advice for screening, diagnosis, and management of fetal growth restriction. Int J Gynaecol Obstet. (2021) 152 Suppl 1:3–57. doi: 10.1002/ijgo.13522 33740264 PMC8252743

[66] Enderle D Spiel A Coticchia CM Berghoff E Mueller R Schlumpberger M. Characterization of RNA from exosomes and other extracellular vesicles isolated by a novel spin column-based method. PloS One. (2015) 10:e0136133. doi: 10.1371/journal.pone.0136133 26317354 PMC4552735

[67] Rozowsky J Kitchen RR Park JJ Galeev TR Diao J Warrell J. exceRpt: A comprehensive analytic platform for extracellular RNA profiling. Cell Syst. (2019) 8:352–7.e3. doi: 10.1016/j.cels.2019.03.004 30956140 PMC7079576

[68] Robinson MD McCarthy DJ Smyth GK. edgeR: a Bioconductor package for differential expression analysis of digital gene expression data. Bioinformatics. (2010) 26:139–40. doi: 10.1093/bioinformatics/btp616 PMC2796818 19910308

[69] Sande AK Torkildsen EA Sande RK Morken NH. Maternal allergy as an isolated risk factor for early-onset preeclampsia: An epidemiological study. J Reprod Immunol. (2018) 127:43–7. doi: 10.1016/j.jri.2018.04.004 29758487

[70] Tarca AL Romero R Bhatti G Gotsch F Done B Gudicha DW. Human plasma proteome during normal pregnancy. J Proteome Res. (2022) 21:2687–702. doi: 10.1021/acs.jproteome.2c00391 PMC10445406 36154181

[71] Kim SH MacIntyre DA Sykes L Arianoglou M Bennett PR Terzidou V. Whole blood holding time prior to plasma processing alters microRNA expression profile. Front Genet. (2021) 12:818334. doi: 10.3389/fgene.2021.818334 35096023 PMC8795683

[72] Meng X Chen C Qian J Cui L Wang S. Energy metabolism and maternal-fetal tolerance working in decidualization. Front Immunol. (2023) 14:1203719. doi: 10.3389/fimmu.2023.1203719 37404833 PMC10315848

[73] Moffett A Shreeve N. Local immune recognition of trophoblast in early human pregnancy: controversies and questions. Nat Rev Immunol. (2023) 23:222–35. doi: 10.1038/s41577-022-00777-2 PMC9527719 36192648

[74] Gainetdinov I Vega-Badillo J Cecchini K Bagci A Colpan C De D. Relaxed targeting rules help PIWI proteins silence transposons. Nature. (2023) 619:394–402. doi: 10.1038/s41586-023-06257-4 37344600 PMC10338343

[75] Jia S Zhang Q Wang Y Wang Y Liu D He Y. PIWI-interacting RNA sequencing profiles in maternal plasma-derived exosomes reveal novel non-invasive prenatal biomarkers for the early diagnosis of nonsyndromic cleft lip and palate. EBioMedicine. (2021) 65:103253. doi: 10.1016/j.ebiom.2021.103253 33639402 PMC7921467

[76] Han H Fan G Song S Jiang Y Qian C Zhang W. piRNA-30473 contributes to tumorigenesis and poor prognosis by regulating m6A RNA methylation in DLBCL. Blood. (2021) 137:1603–14. doi: 10.1182/blood.2019003764 32967010

[77] Than NG Romero R Tarca AL Kekesi KA Xu Y Xu Z. Integrated systems biology approach identifies novel maternal and placental pathways of preeclampsia. Front Immunol. (2018) 9:1661. doi: 10.3389/fimmu.2018.01661 30135684 PMC6092567

[78] Hallal S Ebrahim Khani S Wei H Lee MYT Sim HW Sy J. Deep sequencing of small RNAs from neurosurgical extracellular vesicles substantiates miR-486-3p as a circulating biomarker that distinguishes glioblastoma from lower-grade astrocytoma patients. Int J Mol Sci. (2020) 21:4954. doi: 10.3390/ijms21144954 32668808 PMC7404297

[79] Ma R Liang Z Shi X Xu L Li X Wu J. Exosomal miR-486-5p derived from human placental microvascular endothelial cells regulates proliferation and invasion of trophoblasts via targeting IGF1. Hum Cell. (2021) 34:1310–23. doi: 10.1007/s13577-021-00543-x PMC8338855 33977502

[80] Ghamloush F Ghayad SE Rammal G Fahs A Ayoub AJ Merabi Z. The PAX3-FOXO1 oncogene alters exosome miRNA content and leads to paracrine effects mediated by exosomal miR-486. Sci Rep. (2019) 9:14242. doi: 10.1038/s41598-019-50592-4 31578374 PMC6775163

[81] Hromadnikova I Kotlabova K Hympanova L Krofta L. Cardiovascular and cerebrovascular disease associated microRNAs are dysregulated in placental tissues affected with gestational hypertension, preeclampsia and intrauterine growth restriction. PloS One. (2015) 10:e0138383. doi: 10.1371/journal.pone.0138383 26394310 PMC4579085

[82] Lai W Yu L. Elevated microRNA 183 impairs trophoblast migration and invasiveness by downregulating FOXP1 expression and elevating GNG7 expression during preeclampsia. Mol Cell Biol. (2020) 41:e00236-20. doi: 10.1128/MCB.00236-20 33139493 PMC7849397

[83] Suo M Sun Y Yang H Ji J He Y Dong L. miR-183-5p suppressed the invasion and migration of HTR-8/SVneo trophoblast cells partly via targeting MMP-9 in preeclampsia. Biosci Rep. (2020) 40:BSR20192575. doi: 10.1042/BSR20192575 32342983 PMC7273907

[84] Loscalzo G Scheel J Ibanez-Cabellos JS Garcia-Lopez E Gupta S Garcia-Gimenez JL. Overexpression of microRNAs miR-25-3p, miR-185-5p and miR-132-3p in Late Onset Fetal Growth Restriction, Validation of Results and Study of the Biochemical Pathways Involved. Int J Mol Sci. (2021) 23:293. doi: 10.3390/ijms23010293 35008715 PMC8745308

[85] Qiu Q Tan J. Long noncoding RNA WT1-AS regulates trophoblast proliferation, migration, and invasion via the microRNA-186-5p/CADM2 axis. Open Med (Wars). (2022) 17:1903–14. doi: 10.1515/med-2022-0595 PMC9730544 36561840

[86] Legare C Clement AA Desgagne V Thibeault K White F Guay SP. Human plasma pregnancy-associated miRNAs and their temporal variation within the first trimester of pregnancy. Reprod Biol Endocrinol. (2022) 20:14. doi: 10.1186/s12958-021-00883-1 35031065 PMC8759232

[87] Timofeeva AV Fedorov IS Sukhova YV Ivanets TY Sukhikh GT. Prediction of Early- and Late-Onset Pre-Eclampsia in the Preclinical Stage via Placenta-Specific Extracellular miRNA Profiling. Int J Mol Sci. (2023) 24:8006. doi: 10.3390/ijms24098006 37175711 PMC10178353

[88] Hu S Li J Tong M Li Q Chen Y Lu H. MicroRNA−144−3p may participate in the pathogenesis of preeclampsia by targeting Cox−2. Mol Med Rep. (2019) 19:4655–62. doi: 10.3892/mmr.2019.10150 PMC6522833 31059003

[89] Guo Z Zhu C Wang Y Li Z Wang L Fan J. miR-30a targets STOX2 to increase cell proliferation and metastasis in hydatidiform moles via ERK, AKT, and P38 signaling pathways. Cancer Cell Int. (2022) 22:103. doi: 10.1186/s12935-022-02503-3 35246136 PMC8895545

[90] El-Mogy M Lam B Haj-Ahmad TA McGowan S Yu D Nosal L. Diversity and signature of small RNA in different bodily fluids using next generation sequencing. BMC Genomics. (2018) 19:408. doi: 10.1186/s12864-018-4785-8 29843592 PMC5975555

[91] Chen D Xu L Wu J Liang H Liang Y Liu G. Downregulating miR-96-5p promotes proliferation, migration, and invasion, and inhibits apoptosis in human trophoblast cells via targeting DDAH1. Reprod Biol. (2021) 21:100474. doi: 10.1016/j.repbio.2020.100474 33360846

[92] Zhafir Asyura MMA Komariah M Amirah S Faisal EG Maulana S Platini H. Analysis of varying microRNAs as a novel biomarker for early diagnosis of preeclampsia: A scoping systematic review of the observational study. Int J Prev Med. (2023) 14:36. doi: 10.4103/ijpvm.ijpvm_156_22 37351051 PMC10284242

[93] Deng W Wang X Chen L Wen B Chen Y Ji K. Proteomic and miRNA profiles of exosomes derived from myometrial tissue in laboring women. Int J Mol Sci. (2022) 23:12343. doi: 10.3390/ijms232012343 36293200 PMC9603981

[94] Nikolova M Naydenov M Glogovitis I Apostolov A Saare M Boggavarapu N. Coupling miR/isomiR and mRNA Expression Signatures Unveils New Molecular Layers of Endometrial Receptivity. Life (Basel). (2021) 11:1391. doi: 10.3390/life11121391 34947922 PMC8705090

[95] Xiao J Tao T Yin Y Zhao L Yang L Hu L. miR-144 may regulate the proliferation, migration and invasion of trophoblastic cells through targeting PTEN in preeclampsia. BioMed Pharmacother. (2017) 94:341–53. doi: 10.1016/j.biopha.2017.07.130 28772212

[96] Ogoyama M Takahashi H Suzuki H Ohkuchi A Fujiwara H Takizawa T. Non-coding RNAs and prediction of preeclampsia in the first trimester of pregnancy. Cells. (2022) 11:2428. doi: 10.3390/cells11152428 35954272 PMC9368389

[97] Ning W Chen Y Chen Y Zhang H Wu B Wen C. Correlation and predictive value of serum miR-146b-5p expression during the first trimester of pregnancy with pre-eclampsia. J Obstet Gynaecol. (2022) 42:3537–44. doi: 10.1080/01443615.2022.2153022 36541422

[98] Godoy PM Bhakta NR Barczak AJ Cakmak H Fisher S MacKenzie TC. Large differences in small RNA composition between human biofluids. Cell Rep. (2018) 25:1346–58. doi: 10.1016/j.celrep.2018.10.014 PMC6261476 30380423

[99] McDonough SR Rahman I Sundar IK. Recent updates on biomarkers of exposure and systemic toxicity in e-cigarette users and EVALI. Am J Physiol Lung Cell Mol Physiol. (2021) 320:L661–L79. doi: 10.1152/ajplung.00520.2020 PMC8174828 33501893

[100] Yuan T Huang X Woodcock M Du M Dittmar R Wang Y. Plasma extracellular RNA profiles in healthy and cancer patients. Sci Rep. (2016) 6:19413. doi: 10.1038/srep19413 26786760 PMC4726401

[101] Koch B Gessner A Farmand S Fuhrmann DC Chiocchetti AG Schubert R. Effects of hypoxia on RNA cargo in extracellular vesicles from human adipose-derived stromal/stem cells. Int J Mol Sci. (2022) 23:7384. doi: 10.3390/ijms23137384 35806391 PMC9266528

[102] Huppertz B. Placental physioxia is based on spatial and temporal variations of placental oxygenation throughout pregnancy. J Reprod Immunol. (2023) 158:103985. doi: 10.1016/j.jri.2023.103985 37406413

[103] Burton GJ. Oxygen, the Janus gas; its effects on human placental development and function. J Anat. (2009) 215:27–35. doi: 10.1111/j.1469-7580.2008.00978.x 19175804 PMC2714636

[104] Michlewski G Caceres JF. Post-transcriptional control of miRNA biogenesis. RNA. (2019) 25:1–16. doi: 10.1261/rna.068692.118 30333195 PMC6298569

[105] Fothi A Biro O Erdei Z Apati A Orban TI. Tissue-specific and transcription-dependent mechanisms regulate primary microRNA processing efficiency of the human chromosome 19 MicroRNA cluster. RNA Biol. (2021) 18:1170–80. doi: 10.1080/15476286.2020.1836457 PMC8244754 33052778

[106] Phillips RJ Fortier MA Lopez Bernal A. Prostaglandin pathway gene expression in human placenta, amnion and choriodecidua is differentially affected by preterm and term labour and by uterine inflammation. BMC Pregnancy Childbirth. (2014) 14:241. doi: 10.1186/1471-2393-14-241 25048443 PMC4223419

[107] Zhang D Chang X Bai J Chen ZJ Li WP Zhang C. The study of cyclooxygenase 2 in human decidua of preeclampsia. Biol Reprod. (2016) 95:56. doi: 10.1095/biolreprod.115.138263 27465134

[108] Mirabito Colafella KM Neuman RI Visser W Danser AHJ Versmissen J. Aspirin for the prevention and treatment of pre-eclampsia: A matter of COX-1 and/or COX-2 inhibition? Basic Clin Pharmacol Toxicol. (2020) 127:132–41. doi: 10.1111/bcpt.13308 PMC7496715 31420920

[109] Rolnik DL Nicolaides KH Poon LC. Prevention of preeclampsia with aspirin. Am J Obstet Gynecol. (2022) 226:S1108–S19. doi: 10.1016/j.ajog.2020.08.045 32835720

[110] Rolnik DL Wright D Poon LC O'Gorman N Syngelaki A de Paco Matallana C. Aspirin versus Placebo in Pregnancies at High Risk for Preterm Preeclampsia. N Engl J Med. (2017) 377:613–22. doi: 10.1056/NEJMoa1704559 28657417

[111] Meszaros B Kukor Z Valent S. Recent advances in the prevention and screening of preeclampsia. J Clin Med. (2023) 12:6020. doi: 10.3390/jcm12186020 37762960 PMC10532380

[112] Ju Z Li M Xu J Howell DC Li Z Chen FE. Recent development on COX-2 inhibitors as promising anti-inflammatory agents: The past 10 years. Acta Pharm Sin B. (2022) 12:2790–807. doi: 10.1016/j.apsb.2022.01.002 PMC9214066 35755295

[113] Bishop A Cartwright JE Whitley GS. Stanniocalcin-1 in the female reproductive system and pregnancy. Hum Reprod Update. (2021) 27:1098–114. doi: 10.1093/humupd/dmab028 PMC8542996 34432025

[114] Abid N Embola J Tryfonos Z Bercher J Ashton SV Khalil A. Regulation of stanniocalcin-1 secretion by BeWo cells and first trimester human placental tissue from normal pregnancies and those at increased risk of developing preeclampsia. FASEB J. (2020) 34:6086–98. doi: 10.1096/fj.201902426R PMC7318576 32162740

[115] Juhanson P Rull K Kikas T Laivuori H Vaas P Kajantie E. Stanniocalcin-1 hormone in nonpreeclamptic and preeclamptic pregnancy: clinical, life-style, and genetic modulators. J Clin Endocrinol Metab. (2016) 101:4799–807. doi: 10.1210/jc.2016-1873 PMC5155696 27603899

[116] Cheng JC Chang HM Fang L Sun YP Leung PC. TGF-beta1 up-regulates connexin43 expression: a potential mechanism for human trophoblast cell differentiation. J Cell Physiol. (2015) 230:1558–66. doi: 10.1002/jcp.24902 25560303

[117] Rozas-Villanueva MF Casanello P Retamal MA. Role of ROS/RNS in preeclampsia: are connexins the missing piece? Int J Mol Sci. (2020) 21:4698. doi: 10.3390/ijms21134698 32630161 PMC7369723

[118] Webster RP Roberts VH Myatt L. Protein nitration in placenta - functional significance. Placenta. (2008) 29:985–94. doi: 10.1016/j.placenta.2008.09.003 PMC2630542 18851882

[119] Raguema N Moustadraf S Bertagnolli M. Immune and apoptosis mechanisms regulating placental development and vascularization in preeclampsia. Front Physiol. (2020) 11:98. doi: 10.3389/fphys.2020.00098 32116801 PMC7026478

[120] Rius-Perez S Torres-Cuevas I Millan I Ortega AL Perez S. PGC-1alpha, inflammation, and oxidative stress: an integrative view in metabolism. Oxid Med Cell Longev. (2020) 2020:1452696. doi: 10.1155/2020/1452696 32215168 PMC7085407

[121] Vangrieken P Al-Nasiry S Bast A Leermakers PA Tulen CBM Schiffers PMH. Placental mitochondrial abnormalities in preeclampsia. Reprod Sci. (2021) 28:2186–99. doi: 10.1007/s43032-021-00464-y PMC8289780 33523425

[122] Seremak-Mrozikiewicz A Bogacz A Bartkowiak-Wieczorek J Wolski H Czerny B Gorska-Paukszta M. The importance of MTHFR, MTR, MTRR and CSE expression levels in Caucasian women with preeclampsia. Eur J Obstet Gynecol Reprod Biol. (2015) 188:113–7. doi: 10.1016/j.ejogrb.2015.03.009 25801727

[123] Perez-Sepulveda A Espana-Perrot PP Fernandez XB Ahumada V Bustos V Arraztoa JA. Levels of key enzymes of methionine-homocysteine metabolism in preeclampsia. BioMed Res Int. (2013) 2013:731962. doi: 10.1155/2013/731962 24024209 PMC3762171

[124] Vandewalle A Tourneur E Bens M Chassin C Werts C. Calcineurin/NFAT signaling and innate host defence: a role for NOD1-mediated phagocytic functions. Cell Commun Signal. (2014) 12:8. doi: 10.1186/1478-811X-12-8 24479879 PMC3910266

[125] Kim W Kim E Min H Kim MG Eisenbeis VB Dutta AK. Inositol polyphosphates promote T cell-independent humoral immunity via the regulation of Bruton's tyrosine kinase. Proc Natl Acad Sci USA. (2019) 116:12952–7. doi: 10.1073/pnas.1821552116 PMC6600927 31189594

[126] Merino-Wong M Niemeyer BA Alansary D. Plasma membrane calcium ATPase regulates stoichiometry of CD4(+) T-cell compartments. Front Immunol. (2021) 12:687242. doi: 10.3389/fimmu.2021.687242 34093590 PMC8175910

[127] Huang GN Huso DL Bouyain S Tu J McCorkell KA May MJ. NFAT binding and regulation of T cell activation by the cytoplasmic scaffolding Homer proteins. Science. (2008) 319:476–81. doi: 10.1126/science.1151227 PMC3602998 18218901

[128] Mencarelli A Vacca M Khameneh HJ Acerbi E Tay A Zolezzi F. Calcineurin B in CD4(+) T cells prevents autoimmune colitis by negatively regulating the JAK/STAT pathway. Front Immunol. (2018) 9:261. doi: 10.3389/fimmu.2018.00261 29515579 PMC5826051

[129] Hache S Takser L LeBellego F Weiler H Leduc L Forest JC. Alteration of calcium homeostasis in primary preeclamptic syncytiotrophoblasts: effect on calcium exchange in placenta. J Cell Mol Med. (2011) 15:654–67. doi: 10.1111/j.1582-4934.2010.01039.x PMC3922387 20178461

[130] Halari CD Nandi P Sidhu J Sbirnac M Zheng M Lala PK. Decorin-induced, preeclampsia-associated microRNA-512-3p restrains extravillous trophoblast functions by targeting USF2/PPP3R1 axis. Front Cell Dev Biol. (2022) 10:1014672. doi: 10.3389/fcell.2022.1014672 36299488 PMC9588925

[131] Vernimmen V Paulussen ADC Dreesen J van Golde RJ Zamani Esteki M Coonen E. Preimplantation genetic testing for Neurofibromatosis type 1: more than 20 years of clinical experience. Eur J Hum Genet. (2023) 31:918–24. doi: 10.1038/s41431-023-01404-x PMC10400537 37337089

