# Supplementary figures and images for: Exosomal small RNA profiling in first-trimester maternal blood explores early molecular pathways of preterm preeclampsia
Source: Front Immunol. 2024 Feb 22;15:1321191. doi: 10.3389/fimmu.2024.1321191 (PMC10917917; doi:10.3389/fimmu.2024.1321191)

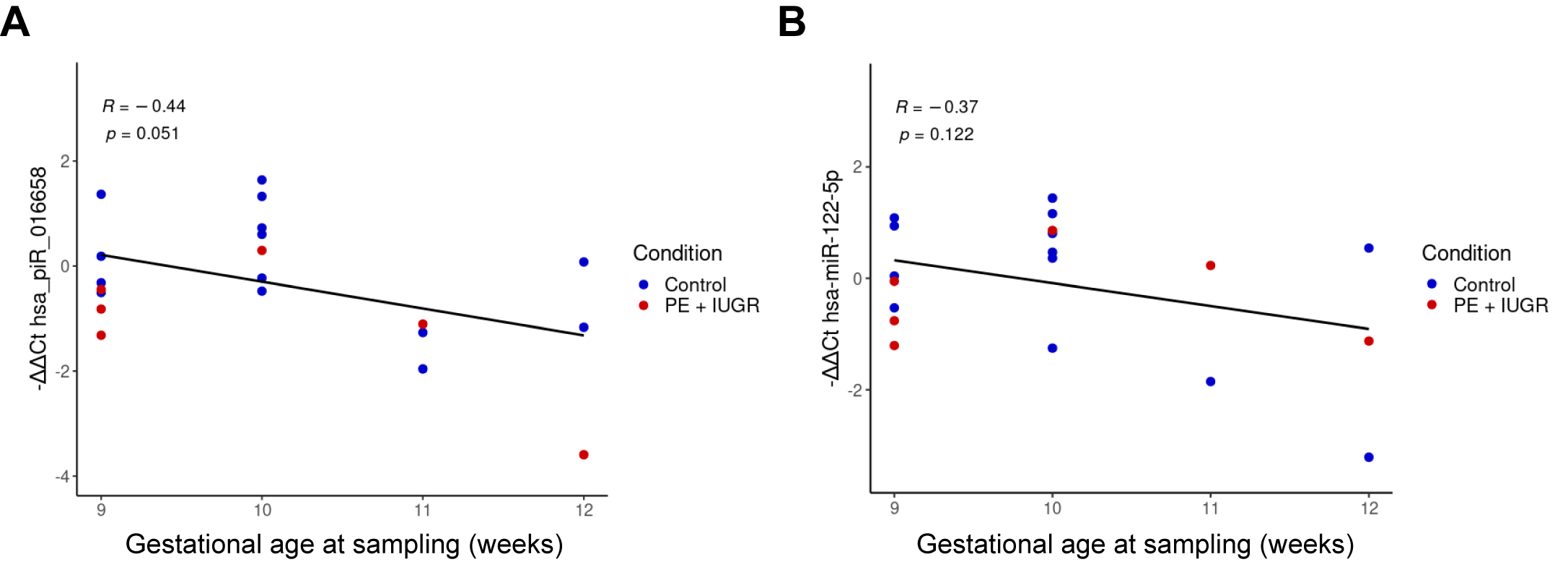

Supplement: Supplementary Figure 1 — Correlation between gestational weeks at sampling time and exosomal small RNA expression measured by qPCR for (A) hsa_piR_016658, p-value=0.051, and (B) hsa-miR-122-5p, p-value=0.122, for PE (n=6, red) and control (n=14, blue) samples. [file Image_1.tif]
