# Supplementary material for: Exosomal small RNA profiling in first-trimester maternal blood explores early molecular pathways of preterm preeclampsia
Source: Front Immunol. 2024 Feb 22;15:1321191. doi: 10.3389/fimmu.2024.1321191 (PMC10917917; doi:10.3389/fimmu.2024.1321191)
Supplement: Supplementary file 2 [file Table_1.docx]

| **Supplementary Table 1.** | | | | | |
| --- | --- | --- | --- | --- | --- |
|  | | | | | |
| **Target mRNAs of differentially expressed exosomal miRNAs and piRNAs**  **in preterm PE with IUGR** | | | | | |
| **Targeted by minimum of n miRNA** | | | | **Targeted by minimum of n piRNA** | |
| **n = 7** | **n = 6** | **n = 5** | **n = 4** | **n = 3** | **n = 2** |
|  |  |  |  |  |  |
| DTNA | ATP2B4 | ARCN1 | AADACL1 | CLN3 | ACBD3 |
|  | CELF2 | BCL11B | ABCC5 | GPATCH2L | ACSBG1 |
|  | CHD6 | CASP2 | ADAR | YAE1 | ACSL6 |
|  | FRS2 | CD46 | AFF4 |  | ARHGAP29 |
|  | METAP2 | CDK12 | AGO1 |  | ARHGDIB |
|  | OTUD4 | CPEB2 | AKAP5 |  | ARR3 |
|  | QKI | ENPP5 | ANKRD12 |  | ATF7IP |
|  | STC1 | GJA1 | ARAP2 |  | BEND5 |
|  | TNRC6A | INTS6 | ASTN1 |  | C2orf15 |
|  |  | KBTBD8 | ATG12 |  | CBX3 |
|  |  | KIAA1737 | ATL1 |  | CC2D2B |
|  |  | KLHL28 | BCL2L11 |  | CCDC125 |
|  |  | NUS1 | BRWD1 |  | CCNH |
|  |  | PCDHA4 | BTBD7 |  | CEP70 |
|  |  | PHF6 | C18orf25 |  | CFAP61 |
|  |  | PHTF2 | C1orf173 |  | CFLAR |
|  |  | PPP2R5C | CA10 |  | CPSF6 |
|  |  | PPP3R1 | CADM2 |  | CYP19A1 |
|  |  | RAP2C | CAMK2D |  | CYP7B1 |
|  |  | REEP3 | CAMK2N1 |  | DHRSX |
|  |  | RNF2 | CCNT2 |  | DISC1 |
|  |  | RNMT | CCP110 |  | DLG2 |
|  |  | SLITRK3 | CDC42BPA |  | DMXL2 |
|  |  | TET1 | CDCA7 |  | EIF2S3 |
|  |  | TPM3 | CDK5R1 |  | EIF5 |
|  |  | YWHAZ | CHD9 |  | EPB41L5 |
|  |  |  | CHIC1 |  | ERCC6L2 |
|  |  |  | CNOT6 |  | ESYT3 |
|  |  |  | CNOT7 |  | ETV1 |
|  |  |  | CPAMD8 |  | F2RL1 |
|  |  |  | CREB5 |  | FBXL4 |
|  |  |  | DCUN1D3 |  | FPGT-TNNI3K |
|  |  |  | DGKB |  | FRMD5 |
|  |  |  | DHDDS |  | GALNT12 |
|  |  |  | DIDO1 |  | GAS2L3 |
|  |  |  | DLC1 |  | GNAI2 |
|  |  |  | DLG5 |  | GNL1 |
|  |  |  | DSTYK |  | GPR151 |
|  |  |  | EDA2R |  | GPR18 |
|  |  |  | ELAVL2 |  | GRIK1 |
|  |  |  | ERC2 |  | GRIN2A |
|  |  |  | ERMN |  | GUCY1A2 |
|  |  |  | EXOC8 |  | HAPSTR1 |
|  |  |  | FAM129A |  | HSBP1 |
|  |  |  | FAM172A |  | IL1RAP |
|  |  |  | FAM91A1 |  | IL7R |
|  |  |  | FAR1 |  | ITM2B |
|  |  |  | FJX1 |  | KCNAB1 |
|  |  |  | FNDC3B |  | KHSRP |
|  |  |  | FOXJ2 |  | KIF4B |
|  |  |  | FRMD6 |  | KPNA4 |
|  |  |  | FSD1L |  | LCOR |
|  |  |  | FYCO1 |  | LCP1 |
|  |  |  | GALNT3 |  | LIN9 |
|  |  |  | GCNT2 |  | MAGI3 |
|  |  |  | GLI3 |  | MAPK9 |
|  |  |  | GNG12 |  | MARK1 |
|  |  |  | GOSR1 |  | MBNL1 |
|  |  |  | HECTD2 |  | MBNL3 |
|  |  |  | HIP1 |  | MRE11 |
|  |  |  | HMGA2 |  | MRPL17 |
|  |  |  | HOMER2 |  | MTR |
|  |  |  | HTR2C |  | MTURN |
|  |  |  | IGF2BP1 |  | MYCN |
|  |  |  | IL1RAP |  | NCKAP1 |
|  |  |  | INTS2 |  | NLGN1 |
|  |  |  | ITGB8 |  | NPY1R |
|  |  |  | JOSD1 |  | NR3C1 |
|  |  |  | KAT6A |  | NRG1 |
|  |  |  | KCNK2 |  | NTNG1 |
|  |  |  | KLF10 |  | NYAP2 |
|  |  |  | KLHL24 |  | OSMR |
|  |  |  | KLHL31 |  | PATE1 |
|  |  |  | LEPROT |  | PAWR |
|  |  |  | LHFPL2 |  | PBOV1 |
|  |  |  | LMO3 |  | PHTF1 |
|  |  |  | LMOD3 |  | POPDC3 |
|  |  |  | MAB21L1 |  | PPARGC1A |
|  |  |  | MAGI3 |  | PPP1R3E |
|  |  |  | MAN1A2 |  | RABGAP1L |
|  |  |  | MAP3K1 |  | RAD54B |
|  |  |  | MAPK1 |  | RASSF3 |
|  |  |  | MAPRE2 |  | RNF34 |
|  |  |  | MEGF9 |  | RPL27A |
|  |  |  | METAP1 |  | SAXO2 |
|  |  |  | MICAL3 |  | SGCE |
|  |  |  | MID1 |  | SIKE1 |
|  |  |  | MIER3 |  | SLC41A2 |
|  |  |  | MPP2 |  | SLC7A6OS |
|  |  |  | MPZL2 |  | SMG6 |
|  |  |  | MYCL1 |  | SNPH |
|  |  |  | NAA30 |  | SPATS1 |
|  |  |  | NAP1L5 |  | SPINK14 |
|  |  |  | NAP5 |  | SPPL2A |
|  |  |  | NCAM1 |  | SPRTN |
|  |  |  | NEUROD1 |  | SPTLC3 |
|  |  |  | NF1 |  | SSTR2 |
|  |  |  | NR4A2 |  | ST6GALNAC3 |
|  |  |  | NTN4 |  | STXBP4 |
|  |  |  | NTRK2 |  | TAS2R42 |
|  |  |  | NXF1 |  | TNPO1 |
|  |  |  | OTUD6B |  | TRIP12 |
|  |  |  | PAK7 |  | TSC22D2 |
|  |  |  | PALM2 |  | UBE2D3 |
|  |  |  | PCGF6 |  | UBE2E2 |
|  |  |  | PEG10 |  | UBE3A |
|  |  |  | PER2 |  | UTP6 |
|  |  |  | PFKFB3 |  | VGLL3 |
|  |  |  | PFN2 |  | WDFY3 |
|  |  |  | PLAGL2 |  | XPR1 |
|  |  |  | PNMA2 |  | YTHDF3 |
|  |  |  | POU4F2 |  | ZNF141 |
|  |  |  | PPP3CA |  | ZNF623 |
|  |  |  | PRKD2 |  | ZNF660 |
|  |  |  | PRRT2 |  | ZNF714 |
|  |  |  | PTGER3 |  |  |
|  |  |  | PTGS2 |  |  |
|  |  |  | PTP4A1 |  |  |
|  |  |  | RAB11FIP1 |  |  |
|  |  |  | RAD23B |  |  |
|  |  |  | RCAN2 |  |  |
|  |  |  | RCHY1 |  |  |
|  |  |  | RIMBP2 |  |  |
|  |  |  | RNF138 |  |  |
|  |  |  | RNF144A |  |  |
|  |  |  | RNF38 |  |  |
|  |  |  | RNGTT |  |  |
|  |  |  | RPRD1A |  |  |
|  |  |  | RRAS2 |  |  |
|  |  |  | S100PBP |  |  |
|  |  |  | SACS |  |  |
|  |  |  | SAR1A |  |  |
|  |  |  | SCN2A |  |  |
|  |  |  | SCN3A |  |  |
|  |  |  | SEL1L |  |  |
|  |  |  | SERP1 |  |  |
|  |  |  | SF1 |  |  |
|  |  |  | SIKE |  |  |
|  |  |  | SIX4 |  |  |
|  |  |  | SLC24A4 |  |  |
|  |  |  | SLC30A8 |  |  |
|  |  |  | SLC35F1 |  |  |
|  |  |  | SLC41A1 |  |  |
|  |  |  | SMAD1 |  |  |
|  |  |  | SNX16 |  |  |
|  |  |  | SOCS6 |  |  |
|  |  |  | SP4 |  |  |
|  |  |  | SPTBN1 |  |  |
|  |  |  | SRGAP2 |  |  |
|  |  |  | SSX1 |  |  |
|  |  |  | ST8SIA2 |  |  |
|  |  |  | SYT10 |  |  |
|  |  |  | TCF7L2 |  |  |
|  |  |  | TMEM56 |  |  |
|  |  |  | TNKS2 |  |  |
|  |  |  | TP53INP1 |  |  |
|  |  |  | TRIM5 |  |  |
|  |  |  | TRPS1 |  |  |
|  |  |  | TSEN15 |  |  |
|  |  |  | TSPYL4 |  |  |
|  |  |  | TUBB |  |  |
|  |  |  | UBE2D1 |  |  |
|  |  |  | UBE3C |  |  |
|  |  |  | UBQLN4 |  |  |
|  |  |  | UBXN7 |  |  |
|  |  |  | USP47 |  |  |
|  |  |  | USP48 |  |  |
|  |  |  | USP6 |  |  |
|  |  |  | WDFY3 |  |  |
|  |  |  | WDR7 |  |  |
|  |  |  | XKRX |  |  |
|  |  |  | YPEL2 |  |  |
|  |  |  | YY1 |  |  |
|  |  |  | ZADH2 |  |  |
|  |  |  | ZBTB10 |  |  |
|  |  |  | ZBTB41 |  |  |
|  |  |  | ZC3H12C |  |  |
|  |  |  | ZC4H2 |  |  |
|  |  |  | ZFP91 |  |  |
|  |  |  | ZNF236 |  |  |
|  |  |  | ZNF280B |  |  |
|  |  |  | ZNF547 |  |  |
|  |  |  | ZNF680 |  |  |
