# Supplementary material for: Exosomal small RNA profiling in first-trimester maternal blood explores early molecular pathways of preterm preeclampsia
Source: Front Immunol. 2024 Feb 22;15:1321191. doi: 10.3389/fimmu.2024.1321191 (PMC10917917; doi:10.3389/fimmu.2024.1321191)
Supplement: Supplementary file 3 [file Table_2.docx]

| **Supplementary Table 2.** | | | | |
| --- | --- | --- | --- | --- |
| **Target mRNAs of differentially expressed small RNAs in preterm PE with IUGR** | | | | |
| **mRNAs targeted by the upregulated**  **miRNAs and/or piRNAs (targeted by minimum n small RNAs)** | | | **mRNAs targeted by the downregulated piRNAs**  **(targeted by minimum n small RNAs)** | |
| **n = 7** | **n = 6** | **n = 5** | **n = 2** | **n = 1** |
|  |  |  |  |  |
| DTNA | ATP2B4 | ANKRD12 | ACSL6 | AAK1 |
| OTUD4 | CELF2 | ARCN1 | BEND5 | AARS2 |
| QKI | CHD6 | BCL11B | CFLAR | ABCA5 |
|  | ENPP5 | CADM2 | DHRSX | ABCC4 |
|  | FRS2 | CASP2 | DLG2 | ABCG5 |
|  | INTS6 | CCNT2 | GAS2L3 | ACACA |
|  | METAP2 | CD46 | GPATCH2L | ACBD3 |
|  | REEP3 | CDK12 | ST6GALNAC3 | ACP6 |
|  | STC1 | CHIC1 | STXBP4 | ACSBG1 |
|  | TNRC6A | CPEB2 |  | ACSM4 |
|  |  | DGKB |  | ACTR1A |
|  |  | DLG5 |  | ACTR5 |
|  |  | FAR1 |  | ADAMTS1 |
|  |  | FYCO1 |  | ADAMTS5 |
|  |  | GALNT3 |  | ADGRF5 |
|  |  | GJA1 |  | ADM5 |
|  |  | HOMER2 |  | AEBP2 |
|  |  | IL1RAP |  | AFM |
|  |  | KBTBD8 |  | AGO1 |
|  |  | KIAA1737 |  | AGPAT4 |
|  |  | KLHL28 |  | AGPAT5 |
|  |  | MAGI3 |  | AK2 |
|  |  | MPZL2 |  | AKR1D1 |
|  |  | NF1 |  | AKT1 |
|  |  | NUS1 |  | ALKBH5 |
|  |  | PCDHA4 |  | ALX3 |
|  |  | PHF6 |  | AMOTL1 |
|  |  | PHTF2 |  | ANKRD18A |
|  |  | PPP2R5C |  | ANKRD18B |
|  |  | PPP3R1 |  | ANKRD33B |
|  |  | PTGER3 |  | ANTXR2 |
|  |  | PTGS2 |  | AP1AR |
|  |  | RAP2C |  | AP1G1 |
|  |  | RNF2 |  | APLP2 |
|  |  | RNMT |  | AQP3 |
|  |  | SCN2A |  | ARAP2 |
|  |  | SF1 |  | ARHGAP20 |
|  |  | SLC24A4 |  | ARHGAP29 |
|  |  | SLITRK3 |  | ARHGAP32 |
|  |  | SPTBN1 |  | ARHGAP44 |
|  |  | TET1 |  | ARHGDIB |
|  |  | TPM3 |  | ARL13B |
|  |  | TRIM5 |  | ARPC5 |
|  |  | WDFY3 |  | ARPP19 |
|  |  | YWHAZ |  | ARR3 |
|  |  |  |  | ARSG |
|  |  |  |  | ARSH |
|  |  |  |  | ASAP1 |
|  |  |  |  | ATF7IP |
|  |  |  |  | ATP5MK |
|  |  |  |  | ATP9A |
|  |  |  |  | B2M |
|  |  |  |  | BAZ1A |
|  |  |  |  | BBS9 |
|  |  |  |  | BCO2 |
|  |  |  |  | BICD1 |
|  |  |  |  | BMAL1 |
|  |  |  |  | BNC2 |
|  |  |  |  | BNIP3 |
|  |  |  |  | BRWD1 |
|  |  |  |  | BTBD2 |
|  |  |  |  | BTF3L4 |
|  |  |  |  | BUD13 |
|  |  |  |  | C1orf185 |
|  |  |  |  | C1orf54 |
|  |  |  |  | C2orf15 |
|  |  |  |  | C2orf49 |
|  |  |  |  | C2orf50 |
|  |  |  |  | CA8 |
|  |  |  |  | CACNA1E |
|  |  |  |  | CACNA2D1 |
|  |  |  |  | CAND1 |
|  |  |  |  | CAPZA2 |
|  |  |  |  | CBFB |
|  |  |  |  | CBL |
|  |  |  |  | CBLL1 |
|  |  |  |  | CBX3 |
|  |  |  |  | CC2D2B |
|  |  |  |  | CCDC125 |
|  |  |  |  | CCDC126 |
|  |  |  |  | CCDC54 |
|  |  |  |  | CCDC93 |
|  |  |  |  | CCNYL1 |
|  |  |  |  | CD302 |
|  |  |  |  | CD44 |
|  |  |  |  | CDC42EP2 |
|  |  |  |  | CDK11A |
|  |  |  |  | CDK11B |
|  |  |  |  | CENPW |
|  |  |  |  | CEP152 |
|  |  |  |  | CEP70 |
|  |  |  |  | CFAP300 |
|  |  |  |  | CFAP52 |
|  |  |  |  | CFAP61 |
|  |  |  |  | CHD9NB |
|  |  |  |  | CHEK1 |
|  |  |  |  | CHRM2 |
|  |  |  |  | CHRM3 |
|  |  |  |  | CHST15 |
|  |  |  |  | CLCC1 |
|  |  |  |  | CLEC18B |
|  |  |  |  | CLN3 |
|  |  |  |  | CLN5 |
|  |  |  |  | CLRN1 |
|  |  |  |  | COA6 |
|  |  |  |  | COL1A2 |
|  |  |  |  | COL4A1 |
|  |  |  |  | COL8A2 |
|  |  |  |  | COPS8 |
|  |  |  |  | CORIN |
|  |  |  |  | COX11 |
|  |  |  |  | CPSF6 |
|  |  |  |  | CREB3L2 |
|  |  |  |  | CRIM1 |
|  |  |  |  | CRX |
|  |  |  |  | CSNK1A1L |
|  |  |  |  | CSPP1 |
|  |  |  |  | CT47B1 |
|  |  |  |  | CTNNB1 |
|  |  |  |  | CTSS |
|  |  |  |  | CYP19A1 |
|  |  |  |  | CYP7B1 |
|  |  |  |  | CYREN |
|  |  |  |  | CYTH1 |
|  |  |  |  | DCAF17 |
|  |  |  |  | DCDC2 |
|  |  |  |  | DCT |
|  |  |  |  | DCUN1D3 |
|  |  |  |  | DDA1 |
|  |  |  |  | DDX52 |
|  |  |  |  | DENND10 |
|  |  |  |  | DERL2 |
|  |  |  |  | DFFA |
|  |  |  |  | DISC1 |
|  |  |  |  | DIXDC1 |
|  |  |  |  | DMAC1 |
|  |  |  |  | DMPK |
|  |  |  |  | DMXL2 |
|  |  |  |  | DNAH12 |
|  |  |  |  | DNAJC14 |
|  |  |  |  | DNAJC21 |
|  |  |  |  | DPM1 |
|  |  |  |  | DPP6 |
|  |  |  |  | DPY19L1 |
|  |  |  |  | DSG2 |
|  |  |  |  | DUOX2 |
|  |  |  |  | DUOXA1 |
|  |  |  |  | DYNLT1 |
|  |  |  |  | DYRK1A |
|  |  |  |  | EBF2 |
|  |  |  |  | EFNA5 |
|  |  |  |  | EFNB2 |
|  |  |  |  | EGLN1 |
|  |  |  |  | EIF1AD |
|  |  |  |  | EIF2S3 |
|  |  |  |  | ELAVL2 |
|  |  |  |  | ELL |
|  |  |  |  | ELOVL7 |
|  |  |  |  | ENO1 |
|  |  |  |  | ENOSF1 |
|  |  |  |  | EPB41 |
|  |  |  |  | EPB41L5 |
|  |  |  |  | EPC1 |
|  |  |  |  | ERBIN |
|  |  |  |  | ERCC6L2 |
|  |  |  |  | ESAM |
|  |  |  |  | ESYT3 |
|  |  |  |  | ETFA |
|  |  |  |  | ETS2 |
|  |  |  |  | ETV1 |
|  |  |  |  | EVC2 |
|  |  |  |  | F2RL1 |
|  |  |  |  | FAM131B |
|  |  |  |  | FAM162A |
|  |  |  |  | FAM174A |
|  |  |  |  | FAM20B |
|  |  |  |  | FAN1 |
|  |  |  |  | FBXL18 |
|  |  |  |  | FBXL4 |
|  |  |  |  | FBXO11 |
|  |  |  |  | FBXO15 |
|  |  |  |  | FBXO30 |
|  |  |  |  | FBXW10B |
|  |  |  |  | FCF1 |
|  |  |  |  | FCMR |
|  |  |  |  | FEZ1 |
|  |  |  |  | FIGN |
|  |  |  |  | FIP1L1 |
|  |  |  |  | FJX1 |
|  |  |  |  | FNIP2 |
|  |  |  |  | FOSL2 |
|  |  |  |  | FOXG1 |
|  |  |  |  | FOXK1 |
|  |  |  |  | FOXL2 |
|  |  |  |  | FOXO3B |
|  |  |  |  | FOXP1 |
|  |  |  |  | FOXP2 |
|  |  |  |  | FPGT |
|  |  |  |  | FPGT-TNNI3K |
|  |  |  |  | FRMD5 |
|  |  |  |  | FRMD8 |
|  |  |  |  | FRMPD3 |
|  |  |  |  | FRS2 |
|  |  |  |  | FSBP |
|  |  |  |  | FUT8 |
|  |  |  |  | GAB3 |
|  |  |  |  | GALC |
|  |  |  |  | GALNT12 |
|  |  |  |  | GANC |
|  |  |  |  | GCC2 |
|  |  |  |  | GCLM |
|  |  |  |  | GCNT3 |
|  |  |  |  | GEN1 |
|  |  |  |  | GET1 |
|  |  |  |  | GET4 |
|  |  |  |  | GIPR |
|  |  |  |  | GLDN |
|  |  |  |  | GLTP |
|  |  |  |  | GNAI1 |
|  |  |  |  | GNAI2 |
|  |  |  |  | GNE |
|  |  |  |  | GNL1 |
|  |  |  |  | GOLGA6B |
|  |  |  |  | GOLGA6C |
|  |  |  |  | GPHN |
|  |  |  |  | GPR151 |
|  |  |  |  | GPR18 |
|  |  |  |  | GPR34 |
|  |  |  |  | GPRC6A |
|  |  |  |  | GRIK1 |
|  |  |  |  | GRIN2A |
|  |  |  |  | GSX2 |
|  |  |  |  | GUCY1A1 |
|  |  |  |  | GUCY1A2 |
|  |  |  |  | GXYLT1 |
|  |  |  |  | H1-0 |
|  |  |  |  | H3-3B |
|  |  |  |  | HAPSTR1 |
|  |  |  |  | HDX |
|  |  |  |  | HECTD1 |
|  |  |  |  | HHIP |
|  |  |  |  | HIGD1A |
|  |  |  |  | HLA-DQA2 |
|  |  |  |  | HMBOX1 |
|  |  |  |  | HNMT |
|  |  |  |  | HNRNPR |
|  |  |  |  | HOXB3 |
|  |  |  |  | HOXD8 |
|  |  |  |  | HP1BP3 |
|  |  |  |  | HS6ST3 |
|  |  |  |  | HSPA2 |
|  |  |  |  | HSPBAP1 |
|  |  |  |  | HTR1E |
|  |  |  |  | IBTK |
|  |  |  |  | IDH3A |
|  |  |  |  | IFI44L |
|  |  |  |  | IFNA5 |
|  |  |  |  | IGF2R |
|  |  |  |  | IL1RAP |
|  |  |  |  | IL31RA |
|  |  |  |  | IL7R |
|  |  |  |  | IMMT |
|  |  |  |  | ING5 |
|  |  |  |  | INPP4B |
|  |  |  |  | INSYN1 |
|  |  |  |  | IRAG2 |
|  |  |  |  | IREB2 |
|  |  |  |  | ITGA11 |
|  |  |  |  | ITM2B |
|  |  |  |  | ITPKB |
|  |  |  |  | ITPRIPL2 |
|  |  |  |  | JADE3 |
|  |  |  |  | JAK1 |
|  |  |  |  | JCAD |
|  |  |  |  | JKAMP |
|  |  |  |  | JMJD4 |
|  |  |  |  | KBTBD6 |
|  |  |  |  | KBTBD8 |
|  |  |  |  | KCNAB1 |
|  |  |  |  | KCNIP4 |
|  |  |  |  | KHSRP |
|  |  |  |  | KIAA0408 |
|  |  |  |  | KIAA2026 |
|  |  |  |  | KIDINS220 |
|  |  |  |  | KIRREL3 |
|  |  |  |  | KLF11 |
|  |  |  |  | KLF6 |
|  |  |  |  | KLHL32 |
|  |  |  |  | KLHL33 |
|  |  |  |  | KLHL5 |
|  |  |  |  | KPNA4 |
|  |  |  |  | KRT6B |
|  |  |  |  | L3MBTL3 |
|  |  |  |  | LAMC3 |
|  |  |  |  | LBR |
|  |  |  |  | LCOR |
|  |  |  |  | LCP1 |
|  |  |  |  | LEPR |
|  |  |  |  | LEPROTL1 |
|  |  |  |  | LETM1 |
|  |  |  |  | LHX9 |
|  |  |  |  | LIN9 |
|  |  |  |  | LOC122319436 |
|  |  |  |  | LPAR4 |
|  |  |  |  | LPP |
|  |  |  |  | LRP1B |
|  |  |  |  | LRRC3B |
|  |  |  |  | LRRC57 |
|  |  |  |  | LRRC70 |
|  |  |  |  | LRSAM1 |
|  |  |  |  | LSM12 |
|  |  |  |  | LY75-CD302 |
|  |  |  |  | LYST |
|  |  |  |  | LZIC |
|  |  |  |  | MAGI3 |
|  |  |  |  | MALRD1 |
|  |  |  |  | MAP10 |
|  |  |  |  | MAP2K3 |
|  |  |  |  | MAP3K1 |
|  |  |  |  | MAP3K20 |
|  |  |  |  | MAPK9 |
|  |  |  |  | MAPKAP1 |
|  |  |  |  | MARK1 |
|  |  |  |  | MBNL1 |
|  |  |  |  | MBNL3 |
|  |  |  |  | MC5R |
|  |  |  |  | MCCC2 |
|  |  |  |  | MCL1 |
|  |  |  |  | MDP1 |
|  |  |  |  | MECOM |
|  |  |  |  | MED12L |
|  |  |  |  | MEF2A |
|  |  |  |  | METTL2A |
|  |  |  |  | METTL2B |
|  |  |  |  | MFN1 |
|  |  |  |  | MFSD4A |
|  |  |  |  | MFSD9 |
|  |  |  |  | MIB1 |
|  |  |  |  | MLF1 |
|  |  |  |  | MMP24OS |
|  |  |  |  | MMP28 |
|  |  |  |  | MOB1B |
|  |  |  |  | MOBP |
|  |  |  |  | MRE11 |
|  |  |  |  | MRGPRX2 |
|  |  |  |  | MRPL17 |
|  |  |  |  | MRPS25 |
|  |  |  |  | MS4A7 |
|  |  |  |  | MSI2 |
|  |  |  |  | MTG2 |
|  |  |  |  | MTO1 |
|  |  |  |  | MTR |
|  |  |  |  | MTURN |
|  |  |  |  | MUC2 |
|  |  |  |  | MYBBP1A |
|  |  |  |  | MYCN |
|  |  |  |  | MYO18A |
|  |  |  |  | MYO7A |
|  |  |  |  | MYOM1 |
|  |  |  |  | MYPOP |
|  |  |  |  | MYRF |
|  |  |  |  | MYRIP |
|  |  |  |  | N4BP2 |
|  |  |  |  | N4BP2L2 |
|  |  |  |  | NAAA |
|  |  |  |  | NAALADL2 |
|  |  |  |  | NAT8L |
|  |  |  |  | NAV2 |
|  |  |  |  | NBEAL1 |
|  |  |  |  | NCAM1 |
|  |  |  |  | NCAPG2 |
|  |  |  |  | NCKAP1 |
|  |  |  |  | NCL |
|  |  |  |  | NCLN |
|  |  |  |  | NCOA5 |
|  |  |  |  | NDFIP1 |
|  |  |  |  | NDST3 |
|  |  |  |  | NDUFAF1 |
|  |  |  |  | NDUFB6 |
|  |  |  |  | NEDD8-MDP1 |
|  |  |  |  | NEFL |
|  |  |  |  | NEPRO |
|  |  |  |  | NF2 |
|  |  |  |  | NHLH2 |
|  |  |  |  | NIPSNAP1 |
|  |  |  |  | NIT2 |
|  |  |  |  | NKAIN1 |
|  |  |  |  | NLGN1 |
|  |  |  |  | NLGN3 |
|  |  |  |  | NNT |
|  |  |  |  | NOX4 |
|  |  |  |  | NPTXR |
|  |  |  |  | NPY1R |
|  |  |  |  | NPY4R |
|  |  |  |  | NPY4R2 |
|  |  |  |  | NR3C1 |
|  |  |  |  | NREP |
|  |  |  |  | NRG1 |
|  |  |  |  | NSMCE3 |
|  |  |  |  | NSUN4 |
|  |  |  |  | NTNG1 |
|  |  |  |  | NUFIP2 |
|  |  |  |  | NUP54 |
|  |  |  |  | NUPR1 |
|  |  |  |  | ODF2 |
|  |  |  |  | OR10H3 |
|  |  |  |  | OR10H5 |
|  |  |  |  | OR13D1 |
|  |  |  |  | OR2AG1 |
|  |  |  |  | OR2J1 |
|  |  |  |  | OR2J2 |
|  |  |  |  | OR2J3 |
|  |  |  |  | OR2L3 |
|  |  |  |  | OR2V1 |
|  |  |  |  | OR4A47 |
|  |  |  |  | OR4C5 |
|  |  |  |  | OR4M2 |
|  |  |  |  | OR4M2B |
|  |  |  |  | OR4Q3 |
|  |  |  |  | OR4S2 |
|  |  |  |  | OR5H2 |
|  |  |  |  | OR6C3 |
|  |  |  |  | OR7E24 |
|  |  |  |  | OR7G2 |
|  |  |  |  | OR8I2 |
|  |  |  |  | OR9Q2 |
|  |  |  |  | OSM |
|  |  |  |  | OSMR |
|  |  |  |  | OTUD7A |
|  |  |  |  | OXNAD1 |
|  |  |  |  | P4HB |
|  |  |  |  | PACRG |
|  |  |  |  | PAFAH1B1 |
|  |  |  |  | PAN3 |
|  |  |  |  | PANK2 |
|  |  |  |  | PANX1 |
|  |  |  |  | PAQR3 |
|  |  |  |  | PARD3 |
|  |  |  |  | PATE1 |
|  |  |  |  | PATL2 |
|  |  |  |  | PAWR |
|  |  |  |  | PAX1 |
|  |  |  |  | PBOV1 |
|  |  |  |  | PCBD2 |
|  |  |  |  | PCDHGB1 |
|  |  |  |  | PCDHGB2 |
|  |  |  |  | PCDHGB6 |
|  |  |  |  | PCDHGB7 |
|  |  |  |  | PCGF5 |
|  |  |  |  | PCYT1B |
|  |  |  |  | PDCL |
|  |  |  |  | PDE11A |
|  |  |  |  | PDE1C |
|  |  |  |  | PDE4A |
|  |  |  |  | PDE7B |
|  |  |  |  | PEX2 |
|  |  |  |  | PF4V1 |
|  |  |  |  | PFKFB3 |
|  |  |  |  | PGGT1B |
|  |  |  |  | PHF12 |
|  |  |  |  | PHLPP2 |
|  |  |  |  | PHTF1 |
|  |  |  |  | PHYHD1 |
|  |  |  |  | PIGA |
|  |  |  |  | PIK3R1 |
|  |  |  |  | PIKFYVE |
|  |  |  |  | PLA2G4F |
|  |  |  |  | PLA2R1 |
|  |  |  |  | PLAA |
|  |  |  |  | PLCG2 |
|  |  |  |  | PNISR |
|  |  |  |  | PNMA5 |
|  |  |  |  | POLE4 |
|  |  |  |  | POLR1B |
|  |  |  |  | POLR2D |
|  |  |  |  | POMT2 |
|  |  |  |  | PON1 |
|  |  |  |  | POPDC3 |
|  |  |  |  | POU3F1 |
|  |  |  |  | PPM1K |
|  |  |  |  | PPP2R3C |
|  |  |  |  | PPP4R3B |
|  |  |  |  | PRKG1 |
|  |  |  |  | PROS1 |
|  |  |  |  | PSMD11 |
|  |  |  |  | PTCD3 |
|  |  |  |  | PTCH1 |
|  |  |  |  | PTHLH |
|  |  |  |  | PTPN20 |
|  |  |  |  | PTPRE |
|  |  |  |  | PURB |
|  |  |  |  | PWWP2A |
|  |  |  |  | RAB39A |
|  |  |  |  | RAD51C |
|  |  |  |  | RAD54B |
|  |  |  |  | RALGAPB |
|  |  |  |  | RALGPS2 |
|  |  |  |  | RAPGEF1 |
|  |  |  |  | RASD2 |
|  |  |  |  | RASGRP3 |
|  |  |  |  | RASSF3 |
|  |  |  |  | RASSF4 |
|  |  |  |  | RC3H1 |
|  |  |  |  | RDM1 |
|  |  |  |  | RELCH |
|  |  |  |  | RETSAT |
|  |  |  |  | RGL4 |
|  |  |  |  | RGS3 |
|  |  |  |  | RHBDD3 |
|  |  |  |  | RIMS1 |
|  |  |  |  | RIOK2 |
|  |  |  |  | RNF144B |
|  |  |  |  | RNF180 |
|  |  |  |  | RNF34 |
|  |  |  |  | RNF41 |
|  |  |  |  | RNPEP |
|  |  |  |  | RNPEPL1 |
|  |  |  |  | RP1L1 |
|  |  |  |  | RPGRIP1L |
|  |  |  |  | RPL14 |
|  |  |  |  | RPL27A |
|  |  |  |  | RPS6KA2 |
|  |  |  |  | RREB1 |
|  |  |  |  | RTN4R |
|  |  |  |  | RUFY3 |
|  |  |  |  | SALL4 |
|  |  |  |  | SAR1A |
|  |  |  |  | SAXO2 |
|  |  |  |  | SBF2 |
|  |  |  |  | SCAPER |
|  |  |  |  | SCD5 |
|  |  |  |  | SCIN |
|  |  |  |  | SCN9A |
|  |  |  |  | SDC4 |
|  |  |  |  | SDCBP |
|  |  |  |  | SDHAF2 |
|  |  |  |  | SEC63 |
|  |  |  |  | SEMA4D |
|  |  |  |  | SERPINA7 |
|  |  |  |  | SESTD1 |
|  |  |  |  | SET |
|  |  |  |  | SGCD |
|  |  |  |  | SGCE |
|  |  |  |  | SH3KBP1 |
|  |  |  |  | SH3RF3 |
|  |  |  |  | SIKE1 |
|  |  |  |  | SIN3A |
|  |  |  |  | SKI |
|  |  |  |  | SLC12A4 |
|  |  |  |  | SLC16A12 |
|  |  |  |  | SLC1A2 |
|  |  |  |  | SLC25A43 |
|  |  |  |  | SLC26A4 |
|  |  |  |  | SLC29A2 |
|  |  |  |  | SLC30A4 |
|  |  |  |  | SLC30A5 |
|  |  |  |  | SLC30A7 |
|  |  |  |  | SLC38A3 |
|  |  |  |  | SLC44A3 |
|  |  |  |  | SLC45A2 |
|  |  |  |  | SLC4A10 |
|  |  |  |  | SLC7A14 |
|  |  |  |  | SLC7A6OS |
|  |  |  |  | SLC9B1 |
|  |  |  |  | SLCO2A1 |
|  |  |  |  | SMARCA2 |
|  |  |  |  | SMARCE1 |
|  |  |  |  | SMC5 |
|  |  |  |  | SMG6 |
|  |  |  |  | SMIM10L1 |
|  |  |  |  | SMOC1 |
|  |  |  |  | SNAP47 |
|  |  |  |  | SNPH |
|  |  |  |  | SNW1 |
|  |  |  |  | SPAST |
|  |  |  |  | SPATS1 |
|  |  |  |  | SPECC1 |
|  |  |  |  | SPINK14 |
|  |  |  |  | SPOCD1 |
|  |  |  |  | SPPL2A |
|  |  |  |  | SPRTN |
|  |  |  |  | SPTLC3 |
|  |  |  |  | SSH1 |
|  |  |  |  | SSTR2 |
|  |  |  |  | STARD4 |
|  |  |  |  | STK25 |
|  |  |  |  | STK32C |
|  |  |  |  | STX16 |
|  |  |  |  | STX6 |
|  |  |  |  | STX7 |
|  |  |  |  | STYX |
|  |  |  |  | SUCNR1 |
|  |  |  |  | SULT1B1 |
|  |  |  |  | SURF4 |
|  |  |  |  | TAF7 |
|  |  |  |  | TAOK3 |
|  |  |  |  | TAS2R42 |
|  |  |  |  | TBC1D13 |
|  |  |  |  | TBCEL |
|  |  |  |  | TBL1X |
|  |  |  |  | TCEAL4 |
|  |  |  |  | TCF7L2 |
|  |  |  |  | TDRD5 |
|  |  |  |  | TENT2 |
|  |  |  |  | TEX13C |
|  |  |  |  | TFDP2 |
|  |  |  |  | TFPI |
|  |  |  |  | TGFBR3 |
|  |  |  |  | THNSL1 |
|  |  |  |  | THOC5 |
|  |  |  |  | THY1 |
|  |  |  |  | TIMELESS |
|  |  |  |  | TIPIN |
|  |  |  |  | TLNRD1 |
|  |  |  |  | TLR6 |
|  |  |  |  | TMCC2 |
|  |  |  |  | TMCO4 |
|  |  |  |  | TMED10 |
|  |  |  |  | TMEM107 |
|  |  |  |  | TMEM183A |
|  |  |  |  | TMEM209 |
|  |  |  |  | TMEM244 |
|  |  |  |  | TMEM248 |
|  |  |  |  | TMEM30B |
|  |  |  |  | TMEM70 |
|  |  |  |  | TMPPE |
|  |  |  |  | TMTC2 |
|  |  |  |  | TNIK |
|  |  |  |  | TNNI1 |
|  |  |  |  | TNPO1 |
|  |  |  |  | TNS3 |
|  |  |  |  | TOR1AIP2 |
|  |  |  |  | TOX3 |
|  |  |  |  | TP73 |
|  |  |  |  | TRA2B |
|  |  |  |  | TRAPPC14 |
|  |  |  |  | TRAPPC5 |
|  |  |  |  | TRIM2 |
|  |  |  |  | TRIM6 |
|  |  |  |  | TRIO |
|  |  |  |  | TRIP12 |
|  |  |  |  | TRNT1 |
|  |  |  |  | TRPC4 |
|  |  |  |  | TSC22D2 |
|  |  |  |  | TTC27 |
|  |  |  |  | TTC3 |
|  |  |  |  | TTC33 |
|  |  |  |  | TUB |
|  |  |  |  | TUBGCP5 |
|  |  |  |  | TXK |
|  |  |  |  | TYMS |
|  |  |  |  | UBA2 |
|  |  |  |  | UBA52 |
|  |  |  |  | UBE2D3 |
|  |  |  |  | UBE2E2 |
|  |  |  |  | UBE2E3 |
|  |  |  |  | UBE3A |
|  |  |  |  | UBR2 |
|  |  |  |  | UFM1 |
|  |  |  |  | UMAD1 |
|  |  |  |  | UPF3B |
|  |  |  |  | USP14 |
|  |  |  |  | USP15 |
|  |  |  |  | USP31 |
|  |  |  |  | USP38 |
|  |  |  |  | USP45 |
|  |  |  |  | USP54 |
|  |  |  |  | UTP6 |
|  |  |  |  | VEZT |
|  |  |  |  | VGLL3 |
|  |  |  |  | VHL |
|  |  |  |  | VKORC1L1 |
|  |  |  |  | VPS13C |
|  |  |  |  | VPS35 |
|  |  |  |  | VPS41 |
|  |  |  |  | VSTM2A |
|  |  |  |  | WDFY3 |
|  |  |  |  | WDHD1 |
|  |  |  |  | XIAP |
|  |  |  |  | XKR5 |
|  |  |  |  | XPR1 |
|  |  |  |  | YAE1 |
|  |  |  |  | YTHDF3 |
|  |  |  |  | ZBTB5 |
|  |  |  |  | ZBTB8A |
|  |  |  |  | ZC3HAV1 |
|  |  |  |  | ZC3HAV1L |
|  |  |  |  | ZC4H2 |
|  |  |  |  | ZCCHC10 |
|  |  |  |  | ZCCHC24 |
|  |  |  |  | ZDHHC13 |
|  |  |  |  | ZDHHC15 |
|  |  |  |  | ZFAND1 |
|  |  |  |  | ZFP36L1 |
|  |  |  |  | ZFX |
|  |  |  |  | ZIC4 |
|  |  |  |  | ZKSCAN2 |
|  |  |  |  | ZKSCAN7 |
|  |  |  |  | ZMAT1 |
|  |  |  |  | ZMYND11 |
|  |  |  |  | ZNF133 |
|  |  |  |  | ZNF141 |
|  |  |  |  | ZNF148 |
|  |  |  |  | ZNF207 |
|  |  |  |  | ZNF215 |
|  |  |  |  | ZNF22 |
|  |  |  |  | ZNF260 |
|  |  |  |  | ZNF274 |
|  |  |  |  | ZNF362 |
|  |  |  |  | ZNF37A |
|  |  |  |  | ZNF454 |
|  |  |  |  | ZNF460 |
|  |  |  |  | ZNF462 |
|  |  |  |  | ZNF518A |
|  |  |  |  | ZNF521 |
|  |  |  |  | ZNF561 |
|  |  |  |  | ZNF577 |
|  |  |  |  | ZNF621 |
|  |  |  |  | ZNF623 |
|  |  |  |  | ZNF649 |
|  |  |  |  | ZNF660 |
|  |  |  |  | ZNF681 |
|  |  |  |  | ZNF714 |
|  |  |  |  | ZNF738 |
|  |  |  |  | ZNF773 |
|  |  |  |  | ZNF891 |
|  |  |  |  | ZXDA |
